# Survival costs of reproduction are independent of energy costs in a seabird, the pelagic cormorant

**DOI:** 10.1002/ece3.11414

**Published:** 2024-07-23

**Authors:** Téo Barracho, Scott A. Hatch, Jana Kotzerka, Stefan Garthe, Hannes A. Schraft, Shannon Whelan, Kyle H. Elliott

**Affiliations:** ^1^ Department of Natural Resource Sciences McGill University Ste‐Anne‐de‐Bellevue Quebec Canada; ^2^ Institute for Seabird Research and Conservation Anchorage Alaska USA; ^3^ Research and Technology Center (FTZ) University of Kiel Buesum Germany; ^4^ Taku River Tlingit First Nation Atlin British Columbia Canada

**Keywords:** activity budgets, capture–mark–recapture, energetics, parental effort, trade‐off

## Abstract

Life‐history theory predicts that investment in reproduction should decrease survival (the ‘cost of reproduction’). It is often assumed that energy allocation drives such trade‐offs, with limited energy available for both reproduction and survival. However, the underlying mechanisms remain poorly understood, maybe because survival costs of reproduction are only apparent when resources are limited. Here, we took advantage of a natural experiment created by fluctuating environmental conditions to compare energy expenditure of a seabird, the pelagic cormorant (*Phalacrocorax pelagicus*), between contrasting population‐scale scenarios of survival costs of reproduction. We used multi‐state capture–recapture modelling across 16 years to identify which breeding seasons induced high survival costs (survival rate_breeders_ < survival rate_non/failed breeders_) and we concomitantly estimated energy expenditure of chick‐rearing males using time‐energy budget models across 4 years. Daily energy expenditure (DEE) of chick‐rearing pelagic cormorants varied significantly among years. However, survival costs of reproduction were observed in only 1 year, and contrary to our expectations, variation in DEE was not associated with population‐level survival costs. Similarly, at the individual level, DEE in 1 year did not predict the probability of being observed again at the colony in following years (apparent survival). Finally, DEE was independent of brood size and brood age, but older individuals tended to expend less energy than younger ones. Given the lack of an apparent energetic ‘cost of reproduction’, lower DEE in older birds could be due to improved efficiency rather than avoidance of costs in old birds. Although future studies should account for potential sex‐specific energetic constraints by including data on female energy expenditure, we conclude that a direct link between the rate of energy expenditure during breeding and subsequent survival is unlikely in this system.

## Graphical abstract

Life‐history theory predicts trade‐offs between survival and reproduction, but underlying mechanisms are seldom investigated. Using a 16‐year long time series of individually monitored pelagic cormorants and 4 years of bio‐logging data, we tested whether increased energy expenditure during reproduction led to reduced survival at both population and individual level. We found no evidence for such a relationship, but older birds tended to expend less energy than younger ones.

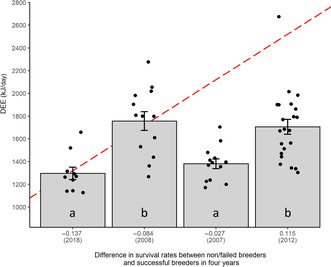

## 1 INTRODUCTION

Life‐history theory assumes the existence of trade‐offs that prevent individuals from maximizing their life history traits as if they were independent (Roff & Fairbairn, 2007; Stearns, 1989, 1992). For example, a trade‐off between current reproduction and survival arises in iteroparous organisms when investment into current reproduction decreases the subsequent probability of survival (the ‘cost of reproduction’, Williams, 1966). Empirical studies that test for this cost have yielded equivocal results (Santos & Nakagawa, 2012) and several potential explanations could explain the inconclusive results. For instance, in experimental studies, where the breeding effort (i.e. workload) of some individuals is artificially modified, the efficacy of the manipulations has been questioned (Boonekamp et al., 2014; Welcker et al., 2015; Williams, 2018). In observational studies that correlate survival and natural breeding effort, the cost may be masked by intra‐population demographic heterogeneity, with some individuals achieving greater reproductive success and survival by acquiring more resources (Cam et al., 1998; van Noordwijk & de Jong, 1986; Weladji et al., 2008). Finally, the relatively sparse evidence for survival costs of reproduction may be due to their dependence on environmental conditions (Robert et al., 2012). Indeed, the trade‐off may be imperceptible when conditions are favourable, and survival costs may be great enough to be observed only when conditions are unfavourable (Barbraud & Weimerskirch, 2005). Unfavourable conditions associated with higher survival costs of reproduction can result from different causes, such as low food availability (Harding et al., 2011; Kitaysky et al., 2010; Williams, 2018), harsh climate (Robert et al., 2012; Tavecchia et al., 2005), disease (Descamps et al., 2009; Garnier et al., 2016) or high population densities (Hamel, Côté, et al., 2010).

But why do difficult environmental conditions increase survival costs of reproduction? Answering this question requires a mechanistic understanding of the survival—reproduction trade‐off (Speakman et al., 2015; Williams, 2018). At the heart of life‐history theory, the allocation principle predicts that survival costs of reproduction arise from greater allocation of energy towards reproduction, to the detriment of self‐maintenance and subsequent survival (Cody, 1966; Stearns, 1992). It is not clear how this imbalanced allocation materializes, and a possibility is that it is in the form of increased energy expenditure during breeding (Golet et al., 2004). Elevated energy expenditure can indeed enhance current breeding success by enabling increased energy transfer towards the offspring (Welcker et al., 2009, 2015), whereas it is often suggested that elevated energy expenditure could decrease subsequent survival through oxidative stress (e.g. Alonso‐Alvarez et al., 2004; but see Speakman et al., 2015; Speakman & Garratt, 2014). Another direct cost of high energy expenditure during reproduction could also be depletion of internal energy reserves (Bryant, 1988), since low body condition is often linked to lower post‐breeding survival (Cornioley et al., 2017; Harding et al., 2011; Welcker et al., 2009). Finally, elevated energy expenditure associated with higher activity rates could also have indirect survival costs, such as increased risks of predation (Magnhagen, 1991) or injury (Guglielmo et al., 2001). Regardless, energy management strategies should balance oxidative stress (i.e., basal metabolic rate, over‐exertion) and food intake (active locomotion; Halsey et al., 2019).

Supporting the hypothesis that unfavourable conditions favour survival costs of reproduction by forcing breeders to expend more energy, daily energy expenditure (DEE) often increases in response to poor conditions during breeding (low food availability: Kitaysky et al., 2000; Regular et al., 2014; Te Marvelde et al., 2011), and high rates of energy expenditure are sometimes associated with lower survival (Daan et al., 1996; Golet et al., 2000). However, despite the compelling argument that energy expenditure should increase under difficult environmental conditions, empirical results are highly varied. For instance, DEE can decrease when food availability is low (Jodice et al., 2006; Welcker et al., 2009), perhaps because energy expenditure is constrained by energy availability (see Figure 3 in Elliott et al., 2014). In other cases, DEE remains remarkably constant across breeding seasons and contrasting environmental conditions (Elliott et al., 2014; Kitaysky et al., 2000; Welcker et al., 2010), which could indicate the existence of an intrinsic ceiling to energy expenditure, independent of food availability and fitness trade‐offs (Elliott et al., 2014). These contrasting results demonstrate that it is difficult to generalize how unfavourable conditions may generate survival costs of reproduction from an energy expenditure perspective. Indeed, it is hard to understand how difficult conditions can produce survival costs of reproduction in populations for which energy expenditure is limited by a physiological ceiling independent of energy availability if these costs arise from increased overall energy expenditure. Interestingly, studies comparing energy expenditure of breeders across contrasting environmental conditions seldom test explicitly for the existence of survival costs of reproduction (i.e. differences in survival between breeders and non‐breeders) in the concerned populations. Thus, when energy expenditure is compared between ‘good’ and ‘poor’ years, we generally do not know whether the conditions termed as ‘poor’ are indeed unfavourable enough to cause the population to suffer survival costs of reproduction. Therefore, simultaneously measuring survival costs of reproduction at the population scale and energy expenditure over several years could help clarify the link between unfavourable conditions, energy expenditure and survival costs of reproduction.

Here, we take advantage of a long‐term monitoring program of pelagic cormorants (*Phalacrocorax pelagicus*) to identify which particular breeding seasons lead to survival costs of reproduction by correlating fitness traits over 16 years (2004–2020), using multi‐state capture–recapture models (Nichols et al., 1994). Concomitantly, we use activity‐specific metabolic rates previously developed at our study site to estimate DEE of chick‐rearing individuals in 4 years based on time budgets derived from GPS loggers or accelerometers. We test the hypothesis that survival costs of reproduction result from an increase in the overall energy expenditure of breeders by comparing DEE among several breeding seasons leading to contrasting scenarios of survival costs at the population level.

We predict that in those years where survival costs of reproduction are apparent (survival rate_breeders_ < survival rate_non‐breeders_), DEE of breeders would be higher than in years without such costs (survival rate_breeders_ ≥ survival rate_non‐breeders_), due to increased time spent in energy‐costly activities (i.e. foraging). We also predict that individuals with low DEE during a given breeding season would have higher apparent survival to the next breeding season, relative to individuals with high DEE. Finally, and to provide further insights into the survival‐reproduction trade‐off, we investigate the effects of DEE on individual breeding productivity.

Although seabirds are generally considered to be on the ‘slow’ side of the slow‐fast continuum of life histories (Gaillard et al., 2016), cormorants can have high annual reproductive output (e.g. up to 4 nestlings per brood), and show important variations in survival and fecundity rates (Frederiksen et al., 2008). Detecting survival costs of reproduction in such species should thus be less challenging compared to other long‐lived seabirds (Hamel, Gaillard, et al., 2010). Furthermore, cormorants operate on low fat stores (income breeding strategy), which makes them highly dependent on acquiring energy through foraging, and thus, through expending energy. Pelagic cormorants, in particular, have the highest absolute flight costs of any bird recorded to date (~20× resting metabolic rate, Elliott et al., 2013), so even minor variations in behaviour are likely to affect their DEE. For these reasons, combined with the availability of longitudinal individual data, pelagic cormorants are a particularly well‐suited model to study the interplay between energy expenditure and survival costs of reproduction.

## 2 MATERIALS AND METHODS

### 2.1 Study species and data collection

Fieldwork was conducted at Middleton Island (59.4°N, 146.3°W), Alaska, USA, where an abandoned radar tower has been converted into artificial seabird nesting habitat. Birds can be observed at each nest from the inside of the tower through one‐way mirrored windows (see Gill & Hatch, 2002 for pictures of the installations). Due to bald eagle (*Haliaeetus leucocephalus*) and glaucous‐winged gull (*Larus glaucescens*) predation of eggs and nestlings, virtually no pelagic cormorants breed successfully away from the tower (gulls and eagles cannot easily access the vertical sites on the tower; Elliott et al., 2011), and there have been very few nestlings produced away from the tower since 2001 despite annual surveys. Thus, the majority of pelagic cormorants on the island nest on this tower and reproductive states are likely to be assessed accurately (i.e. high resighting probability of breeding individuals). Between 2001 and 2020, 643 pelagic cormorants received both a steel band and a numbered plastic band allowing individual identification from inside the tower. The majority of those individuals were banded as adults, with only a small fraction banded as chicks. Banding usually occurred around mid‐July, once nestlings were capable of thermoregulation (about 15 days old). Starting in 2004, resighting and nest monitoring were conducted from inside the tower between early May and mid‐August of each year (except 2014). At the beginning of the breeding season, the identity of all banded individuals was recorded. Later in the season, only nests containing eggs or nestlings were checked every 1–3 days (except 2006–2008 when all nests were checked during the entire field season). At each visit, the number of eggs, number of nestlings, and identity of attending parent(s) (if banded) were recorded. These observations allowed monitoring of individual breeding productivity. Although monitoring focused on breeding individuals, we also observed banded, non‐breeding individuals because they tended to visit the tower, especially at the beginning and the end of the breeding season.

To monitor behaviour, GPS‐pressure loggers (2006–2008: 70 g, dimensions 100 × 48 × 24 mm, Earth & Ocean Technologies, Kiel, Germany; 2015, 2018: 12–30 g GPS, CatTrack 1, Catnip Technologies Ltd., USA; 2018: 3.4 g TDR, LAT1500, Lotek Wireless) were attached to chick‐rearing adults nesting on the tower between June and August (2006: *n* = 14, 2007: *n* = 22, 2008: *n* = 25, 2015: *n* = 7, and 2018: *n* = 28 respectively). The devices recorded time, latitude, longitude and pressure at time intervals between 1 and 330 s, with the exception of 2015, for which pressure data were unavailable. The same was done in 2012 but using accelerometers (CEFAS G6A; 18 g cylinders) instead of GPS (*n* = 22). Over these 6 years, 89 males and 29 females received a logger, although all data could not be used in the analyses (see Section 2.3 below). Birds were captured at their nest using a leg hook and handling time was less than 15 min. Logger deployment occurred at different stages of chick‐rearing (oldest nestling age range: 5–41 days). Device retrieval occurred between 1 and 4 days following initial capture (see Kotzerka et al., 2011 for more details on GPS deployment procedure and Stothart et al., 2016 for accelerometers). Most of the birds equipped with loggers were already banded (68%), but unbanded birds captured for this experiment received a numbered colour band and a steel band. Of the 118 individuals that received a logger (GPS or accelerometer), only 4 (≈3%) failed to fledge at least one nestling, suggesting little impact of capture on breeding success (for comparison, 18.6% of the non‐equipped pairs that hatched eggs failed to fledge at least one nestling over the same six breeding seasons; most failures occurred when nestlings were younger than the age when parents were targeted for tagging). The annual breeding success for the entire tower was calculated as the average number of fledglings per initiated nest (nest where at least one egg was laid).

### 2.2 Modelling variation in survival costs

To determine the years when a high investment in reproduction was associated with a subsequent decrease in survival (‘costly’ years), we considered 2144 observations of individually marked individuals over the 2004–2020 period and used a two‐state classification based on the level of reproductive investment. State 1, reflecting low reproductive investment, included non‐breeders and failed breeders (independently of whether the failure occurred during incubation or chick‐rearing). State 2, reflecting high reproductive investment, included individuals that fledged one or more nestling (successful breeders).

To obtain unbiased estimates of state‐specific survival probabilities, we used multi‐state models for live recaptures, as implemented in program MARK (White & Burnham, 1999). Along with survival probabilities (Φ), multi‐state models compute recapture (*p*) and between‐state transition (ψ) probabilities (Lebreton et al., 2009). We conducted model comparison via Akaike's Information Criterion, corrected for small sample size (AICc, Burnham & Anderson, 2002). Because we were primarily interested in survival rates, we first determined the optimal combination of state, time and sex effects on the parameter of interest for the recapture parameter *p* while maintaining full parametrization (i.e. state, sex and time effects) on Φ and ψ. We then selected the most appropriate *p* structure and used that structure for the comparison of different ψ structures, while maintaining full parametrization on Φ. Finally, we used the best structures for *p* and Ψ to determine the best structure for Φ.

We considered the reproductive event of a year *x* to be costly (i.e. high intensity survival costs) when the probability of survival from year *x* to year *x* + 1 for successful breeders (state 2) was lower than for failed and non‐breeders (state 1). Conversely, we considered the reproductive event of a year *x* to be inexpensive (i.e. low intensity survival costs) in years when survival probabilities of both state 1 and state 2 individuals were indistinguishable. Positive correlations between reproduction and survival, as a result of heterogeneity in quality among individuals, can be strengthened by favourable environmental conditions (Robert et al., 2015). We therefore considered survival costs of reproduction to also be inexpensive in years when survival probabilities of state 2 individuals were found to be higher than for state 1 individuals.

To assess the fit of our model, we tested the fit of a JollyMoVe (JMV) model to the data (Pradel et al., 2003). The JMV model allows survival to vary with time and state of departure, and recapture and transitions to vary with time, previous state and current state (Choquet et al., 2009). Goodness‐of‐fit (GOF) tests were implemented using software U‐CARE (version 2.3.4, Choquet et al., 2009). The overall GOF test revealed some lack of fit of the JMV model to the data (females: *χ* ^2^ = 326.3, df = 170, *p* < .001; males: *χ* ^2^ = 170.8, df = 119, *p* = .001). Inspection of contingency tables and specific tests in U‐CARE revealed that the lack of fit was due to ‘trap‐happiness’ (i.e. a high degree of site fidelity; birds sighted in 1 year were more likely to be resighted the next year than those that had not been seen that year; Test M.ITEC for females: *χ* ^2^ = 143.2, df = 13, *p* < .001; for males: *χ* ^2^ = 75.9, df = 11, *p* < .001; all other tests not significant). Because trap‐dependence can bias parameter estimation (Pradel & Sanz‐Aguilar, 2012), we transformed our data by splitting capture histories and used an age structure on recapture probabilities, following the method of Pradel (1993). Detailed results of GOF tests are provided in the Appendix S1, Table S1.

### 2.3 Estimating energy expenditure

To estimate energy expenditure, we combined time activity budgets extracted from both GPS‐pressure loggers and accelerometers with activity‐specific metabolic rates that had previously been estimated at the same study site using doubly labelled water (Elliott et al., 2013; see Stothart et al., 2016 for the methodology, Table S2 in Appendix S1). To produce activity budgets, we classified behaviour as resting on land, swimming on surface, diving and flying. For GPS, we determined activity budgets using speed, position and pressure. For accelerometers, activity budgets were determined using the depth recorder to record time spent diving and the method of Sakamoto et al. (2009) to determine other activities. Recording length varied between 24 and 72 h. Birds with less than 24 h of data available were not included in the analysis. The methods employed to minimize misinterpretation of behaviour are detailed in the Appendix S1 (Box S1).

We obtained reliable estimates of daily energy expenditure for 71 individuals (10 females and 61 males). Since we did not weigh all individuals, we did not calculate mass‐specific estimates of energy expenditure. Both sexes provide parental care in pelagic cormorants, but since we obtained more data for males, and because females are smaller than males, we excluded females from the analyses. Similarly, data from 2006 (*n* = 2) and 2015 (*n* = 1) were excluded due to low sample sizes in those years. This reduced our final sample size to 58 estimates of DEE, including 13 for 2007, 13 for 2008, 22 for 2012 and 10 for 2018.

### 2.4 Statistical analyses

We investigated inter‐year variation in DEE among 4 years (2007, 2008, 2012, 2018) using linear models. We obtained a small number of repeated DEE measures on individuals (2 years: *n* = 6 birds; 3 years: *n* = 1 bird), so we first fitted linear mixed‐effects models of DEE with a random effect of individual ID (*lme4*, Bates et al., 2014). However, the variance explained by individual ID was consistently estimated as zero, and we therefore continued the analysis using linear models with fixed effects only. We modelled DEE in response to year (four‐level factor), brood age (age of single or oldest nestling at deployment: range = 7–41 days), brood size (number of nestlings at deployment: range = 1–5) and adult age (years since banded: range = 0–15 years). Age since first banding is likely to be a reliable proxy for true age, since it effectively reflects senescence patterns in survival and breeding success in another seabird species (Crespin et al., 2006).

To select the most parsimonious model (i.e. the best combination of covariates and interaction terms), we used R package *MuMIn* (Barton, 2020) to build all possible models nested within the *DEE* ~ *Year* * *Brood size* * *Brood age* * *Adult age* maximal model (with two‐way interactions only) and we compared these models using AICc. We then used the model with the lowest AICc to adjust DEE for the effects of meaningful covariates before testing for differences among years using Tukey‐adjusted pairwise comparisons. There was a degree of multicollinearity in our set of covariates. For example, *Brood age* and *Brood size*, were negatively correlated since brood size tends to decline as the breeding season progresses. As recommended by Cade (2015), we thus chose not to employ model averaging in cases when there was uncertainty in the model selection process (i.e. several models within 2 ΔAICc points of the top model). We instead repeated our analyses on all of the models within 2 ΔAICc points of the top model. We used the same procedure to investigate inter‐year variation in time spent in each activity (flying, diving, resting on land, resting on water).

At the individual level, we tested the effect of DEE in the current season on breeding productivity (number of nestlings fledged, range: 0–4) by modelling breeding productivity in response to DEE and year in a generalized linear model (GLM) with a Poisson distribution (*n* = 58). We examined the effect of DEE during reproduction in the current year on the probability of being seen again at the colony in subsequent years (apparent survival; *n* = 50) and on the probability of breeding successfully (producing at least one fledgling) in the following year (*n* = 37) by using GLMs with logit link function and binomial error structure.

For analyses involving linear models, we checked homoscedasticity and normality of the residuals. If the assumptions were not initially met, we satisfied them by log‐transforming the dependent variable. All statistical analyses except capture–mark–recapture modelling and GOF testing were performed using R (R Core Team, 2016). Means are reported ±1 SE unless specified otherwise.

## 3 RESULTS

### 3.1 Annual breeding success and fluctuations in survival costs of reproduction

**FIGURE 1:**
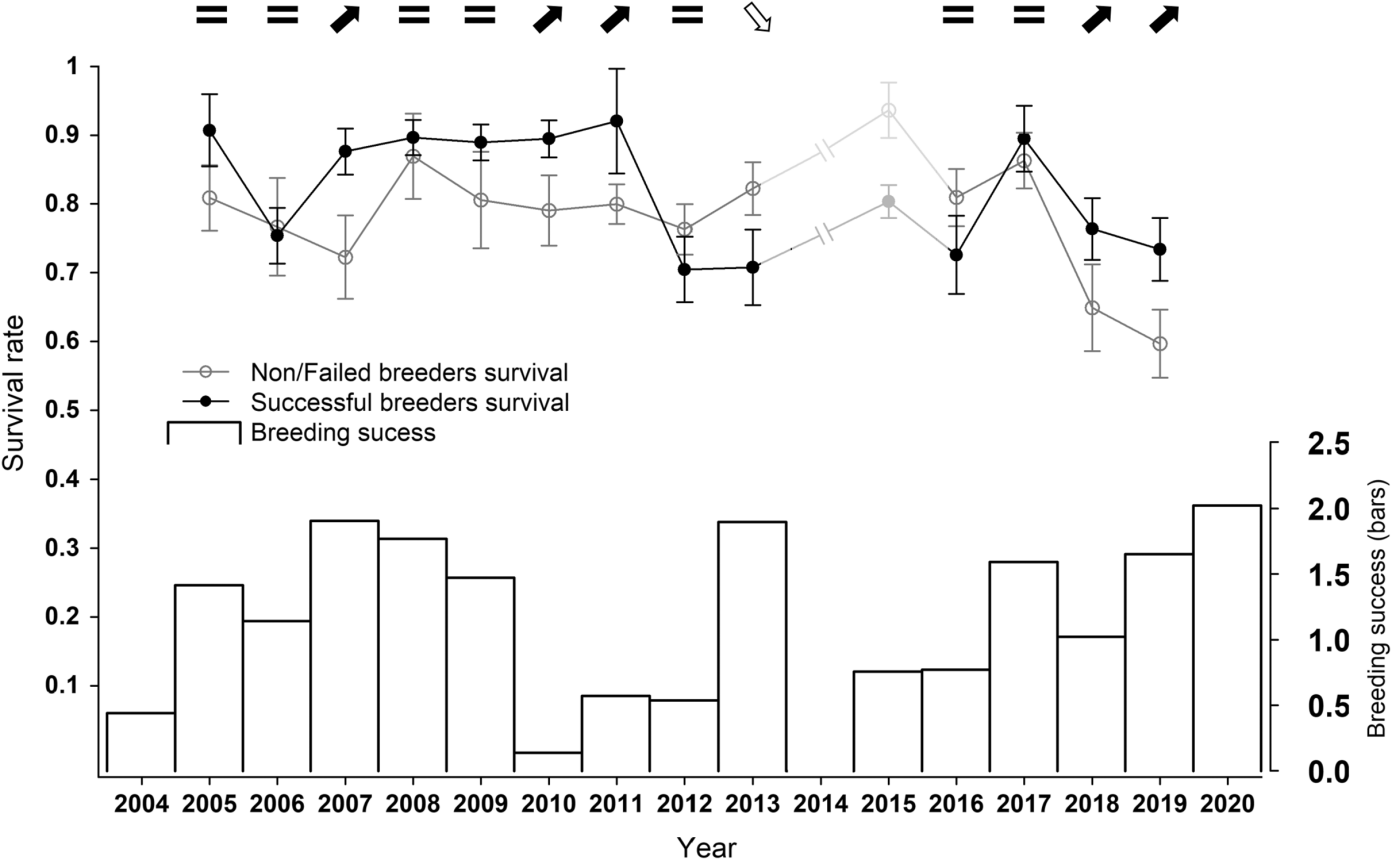
Annual variation in state‐specific survival rates (± SE, black and white circles) and breeding success (bars) for pelagic cormorants on Middleton Island between 2004 and 2020. State‐specific survival probabilities were obtained from the model with the lowest AIC (model 32, Table 1). Each point gives the survival probabilities between year *x*−1 and year *x* as indicated on the *x*‐axis. Survival costs of reproduction were apparent following one breeding season (2012), as indicated by non/failed breeders having a higher survival rate to the next breeding season than successful breeders (white downward arrow). Following other breeding seasons, successful breeders either survived equally well (black equals sign) or better (black upward arrows) than non/failed breeders. No resighting was done in 2014 and survival probabilities for the 2 years interval 2013→2015 (not shown) thus cannot be compared with other estimates. Breeding success (bars) was measured as the average number of fledglings produced per initiated nest (nests in which at least one egg was laid) and includes clutches laid by unbanded individuals.

The average annual number of nesting pairs (i.e. that laid at least one egg) was 84, ranging from 53 in 2016 to 127 in 2008. Between 2004 and 2020, breeding success at the colony varied more than tenfold, ranging from 0.14 fledgling/nest in 2010 to 2.03 fledgling/nest in 2020, with an average of 1.20 (Figure 1, bars). From the 2144 resightings of 643 marked individuals, we obtained 329 unique capture histories, which we used to model state‐specific survival probabilities. Because capture histories were split to accommodate trap‐dependence in our dataset, the model structure for the recapture parameter *p* was constrained to be ‘age’ dependent (age classes = first seen and seen more than once, as recommended by Pradel, 1993). Based on AIC comparisons, the best structure for *p* included state and sex effects for both age classes (Table S3 in Appendix S1). For ψ, the best structure included only time and state effects (Table S3 in Appendix S1), suggesting that sex did not influence the probability of becoming a successful breeder (i.e. entering state 2). For survival probabilities (Φ), the best model included interactive time and state effects (model 32, Table 1). The absence of a sex effect in the best model suggested that the survival of both sexes was similarly affected by reproductive investment (ΔAICc = 14.63 between model 32 and the best ranked model including sex). Model comparisons (Table 1) also showed that the survival of both individuals with high reproductive investment (state 2) and individuals with low reproductive investment (state 1) were more appropriately considered as variable over time rather than constant (ΔAICc = 44.82 between top model and model 26) and that reproductive investment affects survival probabilities (ΔAICc = 15.48 between top model and model 29). Finally, the comparison between model 31 (additive model) and model 32 (top model, including an interaction between state and time) indicated that inter‐annual variations in the survival rates of state 1 and state 2 individuals were not necessarily parallel (Figure 1).

**TABLE 1:** Selection of model structure for survival probabilities (Φ) of pelagic cormorants using AIC corrected for small sample sizes (AICc, Burnham & Anderson, 2002).

Model index	Effect(s) on Φ	Effects on *p* and Ψ	ΔAICc	*w*_*i*_	Np	Deviance
32	Φ (State × Time)	*p* (State × Sex/State × Sex) Ψ (State × Time)	0.00	0.997	68	3732.41
31	Φ (State + Time)	*p* (State × Sex/State × Sex) Ψ (State × Time)	13.41	0.001	54	3774.94
30	Φ (State + Time + Sex)	*p* (State × Sex/State × Sex) Ψ (State × Time)	14.63	0.001	55	3774.10
29	Φ (Time)	*p* (State × Sex/State × Sex) Ψ (State × Time)	15.48	0.000	53	3779.09
28	Φ (Time + Sex)	*p* (State × Sex/State × Sex) Ψ (State × Time)	16.74	0.000	54	3778.28
27	Φ (Time × Sex)	*p* (State × Sex/State × Sex) Ψ (State × Time)	38.71	0.000	68	3771.11
26	Φ (State)	*p* (State × Sex/State × Sex) Ψ (State × Time)	44.82	0.000	40	3835.22
25	Φ (State + Sex)	*p* (State × Sex/State × Sex) Ψ (State × Time)	45.71	0.000	41	3834.06
24	Φ (State × Sex)	*p* (State × Sex/State × Sex) Ψ (State × Time)	47.59	0.000	42	3833.88
23	Φ (State × Time × Sex)	*p* (State × Sex/State × Sex) Ψ (State × Time)	47.66	0.000	98	3716.73
22	Φ (.) (*constant*)	*p* (State × Sex/State × Sex) Ψ (State × Time)	52.91	0.000	39	3845.37
21	Φ (Sex)	*p* (State × Sex/State × Sex) Ψ (State × Time)	53.83	0.000	40	3844.23

*Note*: Best structures for recapture and transition probabilities were previously determined using the same methodology (Table S3 in Appendix S1). The best ranked model (model 32) supports state‐specific survival probabilities with inter‐year variations for each state (i.e. time and state effects on Φ). Np = Number of parameters; ΔAICc = difference in AICc scores between each model and the best model; ×: interaction model; +: additive model.

We were able to interpret state‐specific survival probabilities after all breeding seasons from 2004 to 2018, except 2013 and 2014 (because no resighting occurred in 2014). Survival probabilities following the 2019 breeding season could not be obtained because survival between the penultimate and the last time step cannot be separated from resighting probabilities in the last observational round (2020 in this case, Lebreton et al., 2009).

The state with the highest survival probability varied among years. After seven out of 13 (interpretable) breeding seasons, the survival probabilities of failed and non‐breeders (state 1) were indistinguishable from that of successful breeders (state 2), as indicated by estimates having overlapping standard errors (Figure 1). Following five out of 13 breeding seasons failed and non‐breeders survived less well than successful breeders. In these 12 years, the equal—or better—survival of individuals with high reproductive investment compared to individuals with low investment indicates the low intensity of survival costs of reproduction. Failed and non‐breeders had a higher probability of survival than successful breeders following only one breeding season (2012, Figure 1). Survival costs of reproduction were thus higher in 2012 than in the other years. Specifically, pelagic cormorants that cared for nestlings for the whole chick rearing period in 2012 were ~10% less likely to survive until the 2013 breeding season than individuals that skipped reproduction or did not care for nestlings until fledging in 2012 (S_2012–2013_ state 1 = 0.82 ± 0.04; S_2012–2013_ state 2 = 0.71 ± 0.05). Interestingly, the survival of successful breeders tended to be lower in the latter part of the study (following the 2011 breeding season, mean_pre2011_ = 0.75) compared to earlier years (before 2011, mean_post2011_ = 0.88, Wilcoxon ranks test: *W* = 37, *p* = .022). In contrast, the survival of failed and non‐breeders did not differ significantly between these periods (mean_pre2011_ = 0.79, mean_post2011_ = 0.75, Student *t*‐test: T11 = 1.016, *p* = .331). Parameters estimates for transition and recapture probabilities are presented in the Appendix S1 (Tables S4 and S5).

### 3.2 Variation in DEE

**FIGURE 2:**
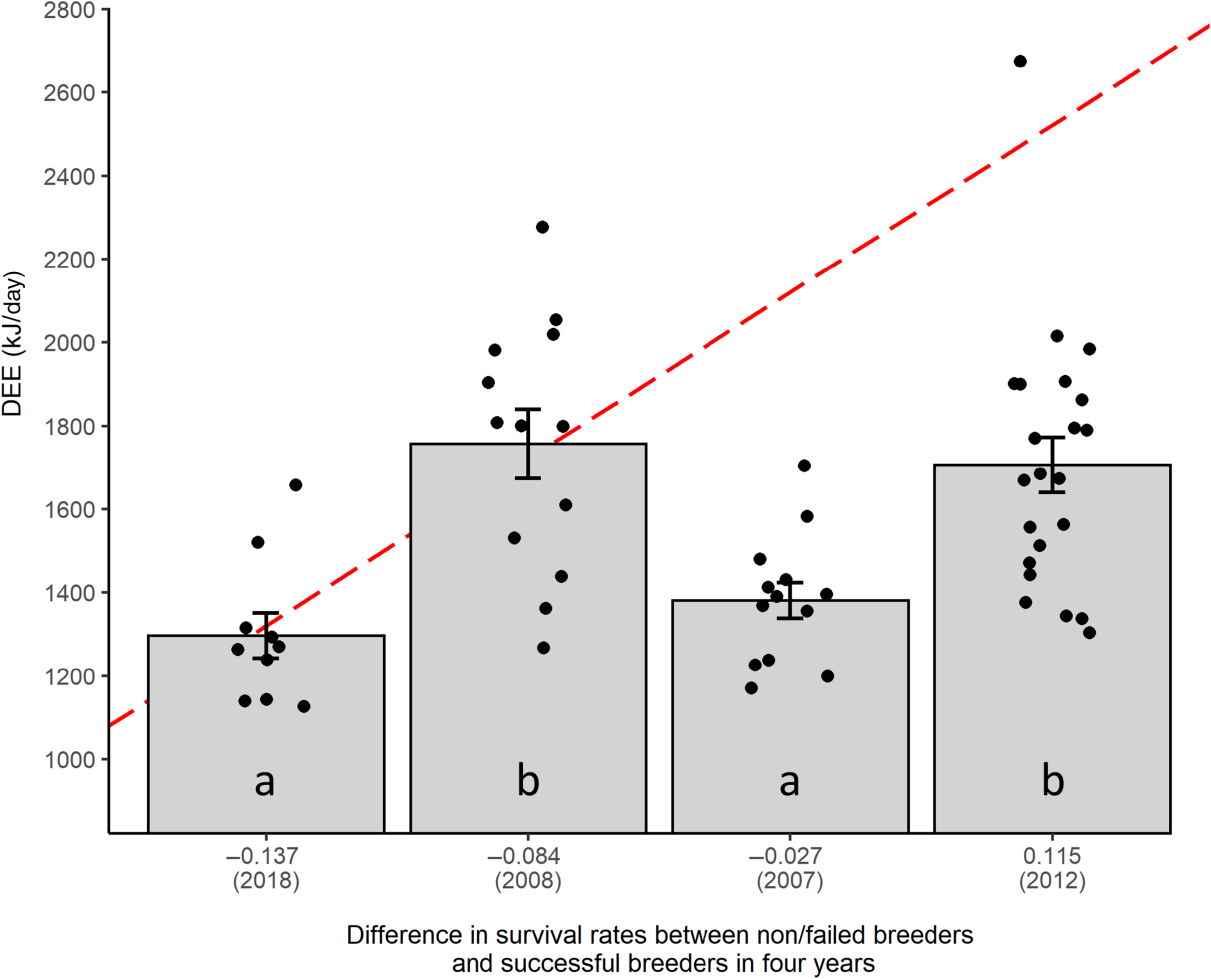
Comparisons of daily energy expenditure (DEE) of male chick rearing pelagic cormorants in 2007, 2008, 2012 and 2018 (mean ± SE, horizontally jittered data points). Groups with the same letter are not statistically different. On the *x*‐axis, a positive difference between the survival rate of non/failed breeders and successful breeders in a given year indicates survival costs of reproduction at the population scale (i.e. successful breeders survived less well than non/failed breeders to the following breeding season). Such costs were apparent only in 2012. DEE in that year was not different from that of 2008, a year where survival costs were not observed. Red dotted line: expected relationship between DEE and cost of reproduction intensity under the hypothesis that survival costs arise from high energy expenditure during breeding.

**FIGURE 3:**
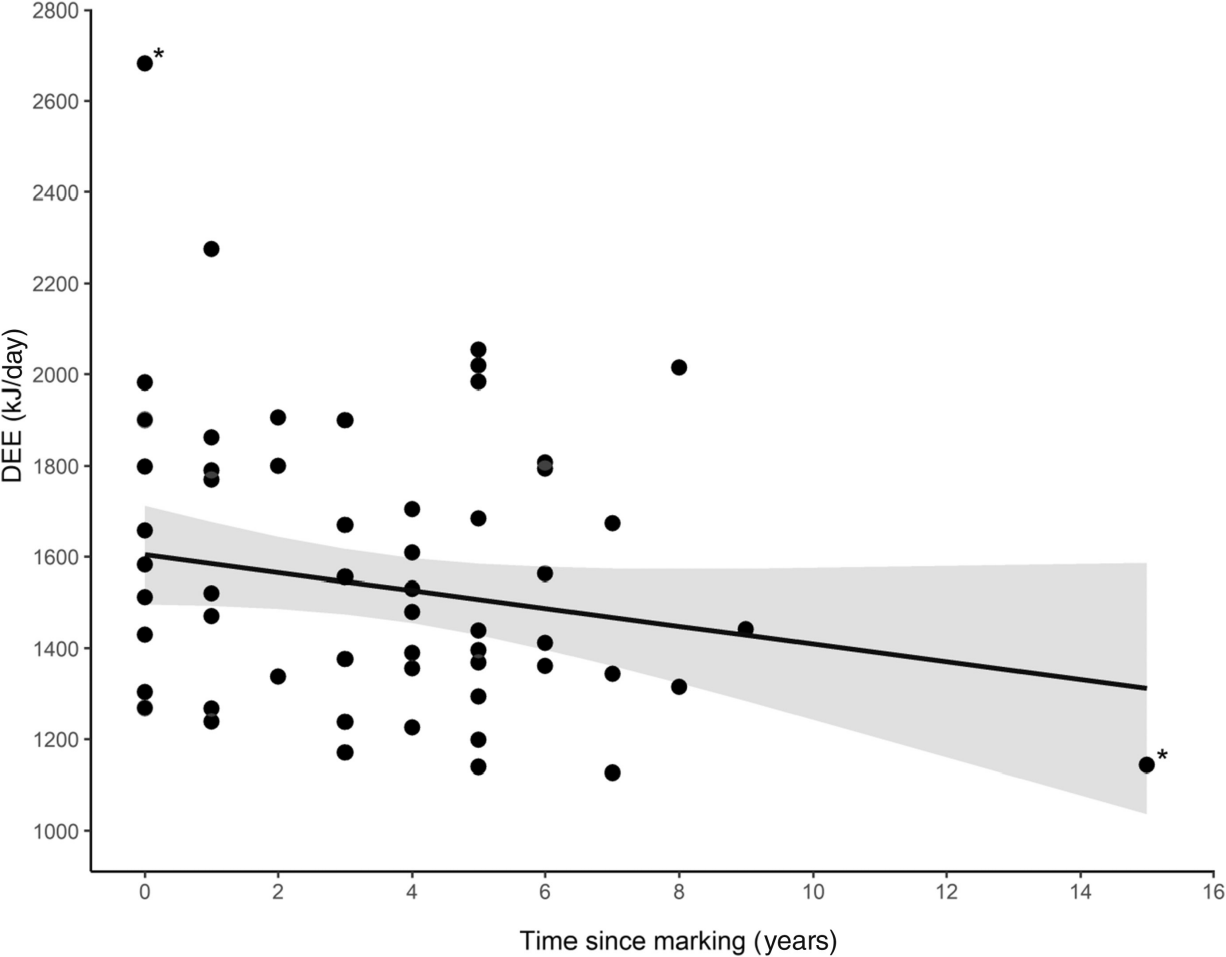
Relationship between daily energy expenditure of chick‐rearing male pelagic cormorants and time since marking, a proxy for true age. Predictions from the best ranked model in Table S6 in Appendix S1 (which also includes an effect of the year) are shown with the black line, with confidence interval in grey. Outliers referred to in the results section are denoted with ‘*’.

Six different models were equally well supported by the data (i.e. within 2 ΔAICc of the best ranked model, Table S6 in Appendix S1). All models included an effect of year, indicating that DEE varied considerably among years. These inter‐annual variations were mainly driven by differences in time spent diving, with birds spending more time diving in the years of higher DEE (Figure S1 and Tables S7–S10 in Appendix S1). We used the best ranked model, which also included an effect of time since marking (model 1, Table S6 in Appendix S1), to perform pairwise comparisons of DEE among years while controlling for time since banding (i.e. by fixing this covariate at its mean). Male pelagic cormorant rearing nestlings expended more energy in 2008 and 2012 (DEE adjusted for time since banding: 1722 ± 72.1 and 1678 ± 53.9 kJ/day respectively) compared to 2007 and 2018 (1376 ± 57.4 and 1301 ± 62.3 kJ/day, respectively, all Tukey‐adjusted *p* < .002, Figure 2). We took model selection uncertainty into account by also performing pairwise comparisons of adjusted DEE among years using the five other models that ranked within 2 ΔAICc of the best ranked model. We obtained similar results for all but one of these models (model 6, Table S6 in Appendix S1), for which the only difference was that DEE in 2007 did not differ from that of 2012 (*t*_52_ = −1.919; Tukey‐adjusted *p* = .233). DEE was lower for older males in all three top models (Figure 3, Table S6 in Appendix S1). However, age was present in only four of the six models ranked within 2 ΔAICc of the best model, suggesting caution in its interpretation. When the model selection process was repeated after excluding two outliers, from the dataset (marked with a ‘*’ in Figure 3), age was included in all models within 2 ΔAICc of the best model. Brood age and brood size did not appear in the top model and were included in only two (brood age) or three (brood size) of the six top models. We thus ignored those effects. In the end, inter‐annual variations in DEE did not match observed patterns of survival costs of reproduction (Figure 2).

### 3.3 Fitness consequences of daily energy expenditure

At the individual level, there was no effect of DEE on current breeding productivity (number of nestlings fledged, $\chi_{1}^{2}$ = 1.213, *p* = .271), and this was similar across all years (interaction term: $\chi_{3}^{2}$ = 0.108, *p* = .991). The number of nestlings fledged nonetheless varied significantly among years ($\chi_{3}^{2}$ = 10.765, *p* = .013). DEE in the current breeding season did not influence apparent survival ($\chi_{1}^{2}$ = 1.321, *p* = .250) or the probability of breeding successfully in the following year ($\chi_{1}^{2}$ = 1.554, *p* = .213).

## 4 DISCUSSION

By measuring DEE in a chick‐rearing seabird while concomitantly assessing variations in vital rates using capture–recapture data, we showed that inter‐annual variations in DEE of male pelagic cormorants were independent of population scale patterns of survival costs of reproduction (Figure 2). Similarly, at the individual level, individuals that died (apparent survival) after a given breeding event had similar energy expenditure than those that survived, and elevated energy expenditure did not appear to enhance current breeding productivity. These findings suggest that the physiological and ecological consequences associated with elevated levels of energy expenditure might not be universal mechanisms underlying the survival‐reproduction life‐history trade‐off.

### 4.1 Inter‐annual variation in survival costs of reproduction

Life‐history theory predicts that the costs of reproduction should occur mainly under unfavourable conditions. When considering long time series, it is thus not expected to observe these costs in every year (Stearns, 1992; Williams, 2018). Here, successful breeders had lower survival than failed and non‐breeders only following the 2012 breeding season, indicating a cost of reproduction on survival. In all other years, the survival of successful breeders was similar to, or higher than, non‐breeders and failed breeders, suggesting that the costs of reproduction in these years were less intense. Greater survival of the individuals that were also the most invested in reproduction (i.e. successful breeders) was observed on five occasions (after the 2006, 2009, 2010, 2017 and 2018 breeding seasons). This pattern of positive covariation between reproduction and survival (Cam et al., 2002; Robert et al., 2015) likely reflects individual heterogeneity in quality or condition (Cam et al., 1998; Lescroël et al., 2009; van Noordwijk & de Jong, 1986; but see Wilson & Nussey, 2010). Because such heterogeneity hampers the detection of fitness trade‐offs (Weladji et al., 2008), we cannot dismiss the existence of survival costs to reproduction in these years. However, there is considerable evidence that unfavourable conditions increase survival costs of reproduction and thus improve our ability to detect them, even when individual heterogeneity is present (Barbraud & Weimerskirch, 2005; Descamps et al., 2009; Garnier et al., 2016; Robert et al., 2012; Tavecchia et al., 2005). In a similar way, favourable conditions promote the observation of positive survival‐reproduction covariations (Robert et al., 2015). Therefore, even if costs of reproduction occur in years of positive survival‐reproduction covariation, cost intensity in those years is likely to be lower than in years where the observed covariation is negative (2012 in this case). In addition, the presence of individual heterogeneity can mask, but not create the appearance of this trade‐off (Sanz‐Aguilar et al., 2008), further reinforcing our interpretation of higher survival costs of reproduction in 2012.

Low food availability is a likely driver of the observed survival costs of reproduction, given the pivotal importance of bottom‐up effects on seabird populations (Cairns, 1987; Kitaysky et al., 2010). First, breeding success in 2012 was low (third lowest in 16 years), and reproductive success often correlates positively with food supply (Cairns, 1987; but see Brisson‐Curadeau et al., 2017). Similarly, survival costs of reproduction have previously been reported only in years of low productivity in another bird species (Plard et al., 2018). Nonetheless, low breeding success was also observed in breeding seasons for which we did not observe survival costs of reproduction (e.g. 2004, 2010). Second, because pelagic cormorants at our study site showed impressive inter‐annual foraging site fidelity during the 2006–2008 period (Kotzerka, 2011; Kotzerka et al., 2011; see Lyons et al., 2007; Peck‐Richardson et al., 2018 for similar behaviour in other marine cormorants), they may be particularly vulnerable to variation in prey abundance or spatial distribution (Kotzerka et al., 2011). More specifically, changes in prey spatial distribution disrupting foraging site fidelity could be a driver of survival costs of reproduction. Unfortunately, no data on foraging locations were available for the only breeding season that led to survival costs of reproduction (2012), preventing a formal testing of this hypothesis. Nonetheless, the survival of individuals highly invested in reproduction (successful breeders) decreased in the latter part of the study (following the 2011 breeding season, Figure 1), and this coincided with an apparent population‐scale diversification of foraging areas (2018 vs. 2007 and 2008, Figure S2 in Appendix S1). This diversification of foraging areas could eventually suggest an actual shift in prey distribution in the latter part of the study. However, the degree of intra‐annual individual foraging site fidelity was similar across years (Figure S2 in Appendix S1), providing little support for this hypothesis. Uncovering the environmental drivers of population scale survival costs of reproduction was not the main aim of the current study, but this question warrants further longitudinal investigations of demographic parameters in conjunction with foraging behaviour (e.g. Lorentsen et al., 2019). In all cases, we ensured that any inter‐annual variations in survival costs of reproduction (independent of their origin) were taken into account by using a time‐dependent parametrization for our estimation of state‐specific survival rates.

### 4.2 A trend for lower DEE in older individuals, but no effects of brood age or size on DEE

Resting metabolic rate tends to decline with increasing age in seabirds, as well as in other animals (Elliott et al., 2015; Moe et al., 2009; but see Moe et al., 2007), but fewer studies investigate age‐related variations in daily energy expenditure. Elliott et al. (2014) reported a positive relationship between DEE and age in handicapped thick‐billed murres (*Uria lomvia*) and suggested that a ‘terminal investment strategy’ could be responsible for this pattern. Our data provided plausible support for lower DEE in older cormorants, which may indicate a negative relationship between age and energy expenditure. This result must however be considered with caution, since time since marking (which we used as a proxy for age) might not perfectly reflect true age. It is unlikely that this pattern resulted from a selective disappearance of individuals with high daily energy expenditure because there was no relationship between the rate of energy expenditure and apparent survival. The lower energy expenditure of older individuals could potentially be explained by age‐related improvements in foraging efficiency (i.e. higher energy intake per energy expenditure), as reported in other seabirds (Le Vaillant et al., 2013), including shags (Daunt et al., 2007). However, such improvements may be mostly effective at early stages (e.g. transition from naïve to experienced breeder), rather than following a linear relationship with age, and it remains unclear whether increasing foraging efficiency can translate into lower energy expenditures. For example, Galbraith et al. (1999) report an increase in foraging efficiency of chick‐rearing common terns (*Sterna hirundo*) with age, but found no relationship between DEE and age. Alternatively, it is also possible that the decrease in DEE with age reflects a decline in physiological ability to expend energy (senescence), as is sometimes proposed for another aspect of metabolism, the resting metabolic rate (Elliott et al., 2015). In other words, older birds may be unable to sustain high rates of activity late in life. Regardless, the reduction in DEE is unlikely to represent an avoidance of costs of reproduction in old birds (e.g. Elliott et al., 2014, 2015) given that we found little evidence for a relationship between energy expenditure and survival.

Interestingly, we did not detect brood age or brood size effects on DEE, the two other covariates included alongside age in our examination of DEE variation among years. Nonetheless, DEE of chick‐rearing parents is sometimes reported to increase with nestling age and number, as adults respond to increasing energy demands (Fyhn et al., 2001; Hicks, Burthe, Daunt, Newell, Butler, et al., 2018; Welcker et al., 2009). Our results could imply that adults were already working near an energy ceiling (Elliott et al., 2014) or that those individuals with larger broods were able to acquire more resources without expending more energy (Welcker et al., 2010; Williams, 2018).

### 4.3 No relationship between daily energy expenditure during breeding and survival

The allocation principle stipulates that survival costs of reproduction arise because of a greater investment of energy towards reproduction at the detriment of survival (Cody, 1966; Stearns, 1992), possibly under the form of high energy expenditure during breeding (Golet et al., 2004). Since unfavourable conditions increase the survival costs of reproduction, it could thus be expected that breeders would expend more energy in poor years compared to good years. Here, the average DEE of chick‐rearing pelagic cormorants differed significantly among years. However, contrary to our initial expectations, these variations did not reflect the observed pattern of survival costs of reproduction (Figure 2). First, DEE did not increase when the relative survival of successful breeders decreased compared to that of non‐breeders and failed breeders (Figure 2). Second, and most importantly, DEE was similar in a year where we observed survival costs (2012) compared to a year where these costs were not observed (2008), even though DEE was higher in 2012 compared to 2007 and 2018 (2 years where survival costs were not observed). Although we were only able to compare a limited number of years (four), this indicates that survival costs of reproduction at the population scale are independent of variation in DEE of breeding individuals. At the individual level, there was no support for a relationship between DEE and survival costs either, with the DEE of individuals that survived a given breeding season being indistinguishable from the DEE of those that did not. This echoes previous findings in little auks (*Alle alle*, Welcker et al., 2009), kittiwakes (*Rissa tridactyla*, Welcker et al., 2010) and thick‐billed murres (*Uria aalge*, Elliott et al., 2014). Overall, our findings (at both the population and individual levels) do not support the idea that high energy expenditure during breeding is driving survival costs of reproduction (Bryant, 1988; Welcker et al., 2009; Williams, 2018). These results may be limited by the absence of energy expenditure data for females since there are often sex‐specific energetic constraints to breeding in seabirds (Hayward & Gillooly, 2011; Hicks, Burthe, Daunt, Newell, Chastel, et al., 2018; Spelt & Pichegru, 2017; Strydom et al., 2023). For example, females are generally considered more flexible in their breeding investment (Spelt & Pichegru, 2017), which could lead them to incur higher energetic costs. Here, male pelagic cormorant show considerable inter‐year variation in energy expenditure (Figure 2), suggesting flexibility in their investment strategy. Furthermore, reproductive investment had similar consequences across sexes in terms of survival (Table 1), which suggests that similar constraints apply to males and females (even though it cannot be excluded that sex‐specific constraints still resulted in the same pattern of survival costs). Together, this makes it less likely for females to display markedly different patterns of energy expenditure during chick‐rearing but warrants future studies where both sexes are included.

### 4.4 Consequences of energy expenditures on current breeding productivity

Long‐term breeding performance is expected to correlate quadratically with energy expenditure (Grémillet et al., 2018), but it is less clear how energy expenditure affects breeding productivity within the current breeding season. For example, Welcker et al. (2009), found a positive relationship in little auks, whereas Kahane‐Rapport et al. (2022) found no support for such links in black‐legged kittiwakes. Here breeding productivity varied among years, but we found no effects of DEE. Yet, elevated activity rates and energy expenditure could have been expected to contribute to maintaining breeding productivity, especially in an unfavourable year that generated survival costs of reproduction (2012). In the end, however, the low breeding success (third lowest in 16 years, Figure 1) and high energy expenditures observed in 2012 (Figure 2) suggest that elevated energy expenditure does not constitute an effective response to maintain breeding productivity when facing unfavourable conditions. This has important implications, as unfavourable years affecting both survival and reproduction are thus more likely to affect the overall population dynamics (Fay et al., 2022).

## 5 CONCLUSIONS

Although initially attractive, the view that high DEE should correlate positively with high survival cost of reproduction continues to lack strong empirical support (but see Daan et al., 1996; Deerenberg et al., 1995). A major gap is that the main mechanism that could link high energy expenditures to lower survival (i.e. a linear and positive relationship between energy expenditure and oxidative damage) is also lacking support (Monaghan et al., 2009; Speakman et al., 2015). Given the tight relationship between energy stores and post‐breeding survival (Cornioley et al., 2017; Harding et al., 2011; Jacobs et al., 2013; Welcker et al., 2009; but see Bryant, 1988), loss of body mass in response to high energy expenditure during breeding is another mechanism that could relate high energy expenditure to lower subsequent survival. However, a coupling between mass loss and energy expenditure may not exist in all species (Bryant, 1988), which could explain the contrasting findings concerning the relationship between energy expenditure and survival. Whether DEE is linked to body mass could potentially depend on how close to their physiological energetic ceiling (sensu Elliott et al., 2014) individuals have to work during breeding. DEE and mass loss could correlate positively if individuals working beneath their energy ceiling mobilize reserves to increase DEE when conditions deteriorate. On the other hand, individuals already operating at or near their energy ceiling when conditions are good will be unable to compensate for a decrease in food availability by increasing energy expenditure and foraging behaviour. Under a reproductive costs scenario (i.e. costs are not passed to offspring), individuals would preferentially invest the reduced amount of energy available in their progeny, therefore not replenishing their own body reserves. In this case, DEE and mass loss would not correlate, or could even correlate negatively if individuals with a higher ceiling are able to use the extra energy they can expend to exploit better foraging sites and gather enough energy to replete their reserves to some extent (thus losing less mass). However, as cormorants have very little body reserves compared to most other seabirds, this explanation seems unlikely to apply to our study species.

In summary, our study indicates that a direct relationship between DEE during breeding and subsequent survival is unlikely in pelagic cormorants. Still, a possible limitation is that our energy expenditure estimates were based solely on movement, although other mechanisms such as thermoregulation (Grémillet & Wilson, 1999) or parasitism (Hicks, Burthe, Daunt, Newell, Butler, et al., 2018; Hicks, Burthe, Daunt, Newell, Chastel, et al., 2018) could affect DEE. As an alternative to direct negative consequences of energy expenditure, the survival cost to reproduction we observed in 1 year (2012) could be explained by breeders being unable to replenish their internal energy stores after breeding, if mass loss is independent from energy expenditure. We did not investigate changes in body mass, but testing the relationship between DEE and mass loss during breeding could provide explanations for the contrasting results from the studies investigating the relationship between energy expenditure and survival. Specifically, high DEE during breeding might be associated with lower subsequent survival only when body mass decreases with increasing DEE.

## AUTHOR CONTRIBUTIONS

**Téo Barracho:** Conceptualization (supporting); data curation (equal); formal analysis (lead); methodology (equal); validation (equal); writing – original draft (lead); writing – review and editing (equal). **Scott A. Hatch:** Conceptualization (equal); data curation (equal); funding acquisition (equal); investigation (equal); methodology (equal); project administration (lead); resources (equal); validation (equal); writing – review and editing (equal). **Jana Kotzerka:** Data curation (equal); investigation (equal); validation (equal); writing – review and editing (equal). **Stefan Garthe:** Data curation (equal); investigation (equal); validation (equal); writing – review and editing (equal). **Hannes A. Schraft:** Data curation (equal); investigation (equal); validation (equal); writing – review and editing (equal). **Shannon Whelan:** Data curation (equal); formal analysis (supporting); investigation (equal); validation (equal); writing – review and editing (equal). **Kyle H. Elliott:** Conceptualization (lead); data curation (equal); formal analysis (equal); funding acquisition (lead); investigation (equal); methodology (equal); project administration (lead); resources (equal); supervision (lead); validation (equal); writing – original draft (lead); writing – review and editing (equal).

## CONFLICT OF INTEREST STATEMENT

The authors declare no conflicts of interest.

### OPEN RESEARCH BADGES

This article has earned an Open Data badge for making publicly available the digitally‐shareable data necessary to reproduce the reported results. The data is available at https://doi.org/10.5061/dryad.t76hdr882.

## Supporting information


Appendix S1.

## Data Availability

Data will be made available on Dryad conditional on the acceptance of the paper.
