# Supplementary material for: Survival costs of reproduction are independent of energy costs in a seabird, the pelagic cormorant
Source: Ecol Evol. 2024 Jul 23;14(7):e11414. doi: 10.1002/ece3.11414 (PMC11264352; doi:10.1002/ece3.11414)
Supplement: Supplementary file 1 — Appendix S1. [file ECE3-14-e11414-s001.zip › appendix.docx]

**Appendix:**

**I-Tables and figures**

**Table S1.** Results of Goodness-of-fit tests. Df = Degrees of freedom; ĉ = Deviance inflation factor.

| Group 1 (females) | |  |  |  |
| --- | --- | --- | --- | --- |
| Test | χ^2^ | P-value | Df | ĉ =χ2/Df |
| WBWA | 34.57 | 0.183 | 28.00 | 1.23 |
| 3G.SR | 31.82 | 0.282 | 28.00 | 1.14 |
| 3G.Sm | 107.37 | 0.102 | 90.00 | 1.19 |
| M.ITEC | 143.25 | <0.001 | 13.00 | 11.02 |
| M.LTEC | 9.29 | 0.595 | 11.00 | 0.84 |
| Sum | 326.30 | <0.001 | 170.00 | 1.92 |
|  |  |  |  |  |
| Group 2 (males) | |  |  |  |
| Test | χ^2^ | P-value | Df | ĉ =χ^2^/Df |
| WBWA | 18.75 | 0.809 | 25.00 | 0.75 |
| 3G.SR | 12.12 | 0.99 | 26.00 | 0.47 |
| 3G.Sm | 64.03 | 0.244 | 57.00 | 1.12 |
| M.ITEC | 75.94 | <0.001 | 11.00 | 6.90 |
| M.LTEC | Na | Na | Na | Na |
| Sum | 170.83 | 0.001 | 119.00 | 1.44 |

**Table S2.** Activity-specific energy expenditures in Watts (Joules per second) for different types of activities, from Elliott *et al.* 2013 (see reference in the main text). These estimations come from a doubly labelled water experiment realized in 2012 at our study site, using accelerometers to measure behaviour. Detailed calculations of activity specific energy expenditures can be found in Stothart *et al.* 2016.

| Activity : | Flying | Diving | Resting (water) | Resting (land) |
| --- | --- | --- | --- | --- |
| Specific metabolic rate (W) : | 168 ± 51 | 66 ± 11 | 7.7 ± 12 | 7.8 ± 2.5 |

**Table S3.** Model selection for the structure of recapture probabilities (*p*) and between-state transition probabilities (Ψ). Because we were mainly interested in survival probabilities, we first determined the best structures for *p* (models 1 to 8), then for Ψ (models 9 to 20), before using these structures to determine the best structure for the survival probability Φ. To maximize parameters estimability, interaction models (×) were built using a sin link function, and additive models (+) using a log link function, as implemented in MARK (White and Burnham 1999). The best ranked model for recapture probabilities (model 8) includes two age classes, with state and sex effects on the recapture probabilities for both the first age class (birds seen once) and the second age class (birds seen more than once). The best ranked model for transition probabilities (model 20) includes interactive state and time effects, with no effect of sex. Np = Number of parameters ; ΔAICc = difference in AICc scores between each model and the best model of each set of comparisons; *w_i_* = Akaike weights.

| **Index** | **Model** | **Effect(s) on *p* or Ψ** | **ΔAICc** | ***w_i_*** | **Np** | **Deviance** |
| --- | --- | --- | --- | --- | --- | --- |
|  |  |  |  |  |  |  |
| Model selection for recapture probabilities (p) | |  |  |  |  |  |
| 8 | { Φ(State × Time × Sex) p(State × Sex/State × Sex) Ψ(State × Time × Sex)} | 2 age classes (1 and 1+), p _1_ varies with state and sex, p_1+_ varies with state and sex | 0.00 | 0.99 | 128 | 3700.15 |
| 7 | { Φ(State × Time × Sex) p(Sex/Sex) Ψ(State × Time × Sex)} | 2 age classes (1 and 1+), p _1_ varies with sex, p_1+_ varies with sex | 8.62 | 0.01 | 124 | 3717.46 |
| 6 | { Φ(State × Time × Sex) p(State/State) Ψ(State × Time × Sex)} | 2 age classes (1 and 1+), p _1_ varies with state, p_1+_ varies with state | 30.03 | 0.00 | 124 | 3738.86 |
| 5 | { Φ(State × Time × Sex) p(./.) Ψ(State × Time × Sex)} | 2 age classes (1 and 1+), p _1_ constant, p_1+_ constant | 44.07 | 0.00 | 122 | 3757.24 |
| 4 | { Φ(State × Time × Sex) p(Time × Sex/Time × Sex) Ψ(State × Time × Sex)} | 2 age classes (1 and 1+), p _1_ varies with time and sex, p_1+_ varies with time and sex | 49.20 | 0.00 | 173 | 3650.01 |
| 3 | { Φ(State × Time × Sex) p(Time/Time) Ψ(State × Time × Sex)} | 2 age classes (1 and 1+), p _1_ varies with time, p_1+_ varies with time | 57.22 | 0.00 | 149 | 3711.38 |
| 2 | { Φ(State × Time × Sex) p(State × Time/State × Time) Ψ(State × Time × Sex)} | 2 age classes (1 and 1+), p _1_ varies with state and time, p_1+_ varies with state and time | 62.80 | 0.00 | 176 | 3656.88 |
| 1 | { Φ(State × Time × Sex) p(State × Time × Sex/State × Time × Sex) Ψ(State × Time × Sex)} | 2 age classes (1 and 1+), p _1_ varies with state, time and sex, p_1+_ varies with state, time and sex | 124.20 | 0.00 | 230 | 3594.76 |
|  |  |  |  |  |  |  |
| Model selection for transition probabilities (Ψ) | |  |  |  |  |  |
| 20 | {Φ(State × Time × Sex) p(State × Sex/State × Sex) Ψ(State × Time)} | Ψ varies with state and time (interaction model) | 0.00 | 1.00 | 98.00 | 3716.73 |
| 19 | {Φ(State × Time × Sex) p(State × Sex/State × Sex) Ψ(State × Time × Sex)} | Ψ varies with state, time and sex (interaction model) | 48.01 | 0.00 | 128.00 | 3700.15 |
| 18 | {Φ(State × Time × Sex) p(State × Sex/State × Sex) Ψ(Time)} | Ψ varies with time | 382.98 | 0.00 | 83.00 | 4131.53 |
| 17 | {Φ(State × Time × Sex) p(State × Sex/State × Sex) Ψ(State + Time)} | Ψ varies with state and time (additive model) | 383.51 | 0.00 | 84.00 | 4129.95 |
| 16 | {Φ(State × Time × Sex) p(State × Sex/State × Sex) Ψ(Time + Sex)} | Ψ varies with time and sex (additive model) | 383.90 | 0.00 | 84.00 | 4130.34 |
| 15 | {Φ(State × Time × Sex) p(State × Sex/State × Sex) Ψ(State + Time + Sex)} | Ψ varies with state, time and sex (additive model) | 384.37 | 0.00 | 85.00 | 4128.70 |
| 14 | {Φ(State × Time × Sex) p(State × Sex/State × Sex) Ψ(Time × Sex)} | Ψ varies with time and sex (interaction model) | 408.04 | 0.00 | 98.00 | 4124.77 |
| 13 | {Φ(State × Time × Sex) p(State × Sex/State × Sex) Ψ(State × Sex)} | Ψ varies with state and sex (interaction model) | 492.34 | 0.00 | 72.00 | 4264.02 |
| 12 | {Φ(State × Time × Sex) p(State × Sex/State × Sex) Ψ(.)} | Ψ constant | 494.46 | 0.00 | 69.00 | 4272.43 |
| 11 | {Φ(State × Time × Sex) p(State × Sex/State × Sex) Ψ(Sex)} | Ψ varies with sex | 495.36 | 0.00 | 70.00 | 4271.24 |
| 10 | {Φ(State × Time × Sex) p(State × Sex/State × Sex) Ψ(State)} | Ψ varies with state | 496.40 | 0.00 | 70.00 | 4272.28 |
| 9 | {Φ(State × Time × Sex) p(State × Sex/State × Sex) Ψ(State + Sex)} | Ψ varies with state and sex (additive model) | 497.30 | 0.00 | 71.00 | 4271.08 |

**Table S4.** Estimates of state transition probabilities from model 32 (top model, Table 1, main text). The state transition probability in year x corresponds to the probability for an individual that was in a given state in year x-1 to enter the other state in year x. For example, an individual in state 1 in 2004 had a probability of 0.609 to enter state 2 in 2005.

SE = Standard Error ; LCI = Lower Confidence Interval ; UCI = Upper Confidence Interval

| Probability of transition from state 1 to state 2 | | | | |  | Probability of transition from state 2 to state 1 | | | | |
| --- | --- | --- | --- | --- | --- | --- | --- | --- | --- | --- |
| **Year** | **Estimate** | **SE** | **LCI** | **UCI** |  | **Year** | **Estimate** | **SE** | **LCI** | **UCI** |
| 2005 | 0.609 | 0.062 | 0.483 | 0.722 |  | 2005 | 0.142 | 0.059 | 0.060 | 0.298 |
| 2006 | 0.488 | 0.096 | 0.310 | 0.670 |  | 2006 | 0.440 | 0.053 | 0.341 | 0.545 |
| 2007 | 0.786 | 0.067 | 0.626 | 0.889 |  | 2007 | 0.229 | 0.043 | 0.156 | 0.323 |
| 2008 | 0.881 | 0.086 | 0.597 | 0.974 |  | 2008 | 0.220 | 0.033 | 0.162 | 0.292 |
| 2009 | 0.554 | 0.106 | 0.348 | 0.742 |  | 2009 | 0.405 | 0.039 | 0.332 | 0.482 |
| 2010 | 0.021 | 0.020 | 0.003 | 0.129 |  | 2010 | 0.929 | 0.019 | 0.881 | 0.958 |
| 2011 | 0.312 | 0.034 | 0.250 | 0.382 |  | 2011 | 0.296 | 0.121 | 0.119 | 0.566 |
| 2012 | 0.273 | 0.043 | 0.196 | 0.365 |  | 2012 | 0.494 | 0.060 | 0.379 | 0.609 |
| 2013 | 0.782 | 0.045 | 0.681 | 0.858 |  | 2013 | 0.113 | 0.046 | 0.049 | 0.239 |
| 2015 | 0.118 | 0.076 | 0.031 | 0.357 |  | 2015 | 0.657 | 0.046 | 0.561 | 0.742 |
| 2016 | 0.278 | 0.049 | 0.193 | 0.383 |  | 2016 | 0.460 | 0.072 | 0.325 | 0.600 |
| 2017 | 0.457 | 0.054 | 0.354 | 0.563 |  | 2017 | 0.378 | 0.069 | 0.255 | 0.519 |
| 2018 | 0.273 | 0.070 | 0.158 | 0.428 |  | 2018 | 0.425 | 0.055 | 0.323 | 0.535 |
| 2019 | 0.477 | 0.065 | 0.354 | 0.602 |  | 2019 | 0.238 | 0.048 | 0.156 | 0.344 |
| 2020 | 0.459 | 0.096 | 0.284 | 0.645 |  | 2020 | 0.245 | 0.044 | 0.169 | 0.340 |
|  |  |  |  |  |  |  |  |  |  |  |

**Table S5.** Estimates of state, sex, and age class –specific recapture probabilities from model 32 (top model, Table 1, main text).

SE = Standard Error ; LCI = Lower Confidence Interval ; UCI = Upper Confidence Interval

| **State** | **Sex** | **Age group** | **Estimate** | **SE** | **LCI** | **UCI** |
| --- | --- | --- | --- | --- | --- | --- |
| 1 | Female | 1 | 0,825 | 0,024 | 0,773 | 0,867 |
| 1 | Male | 1+ | 0,409 | 0,056 | 0,306 | 0,520 |
| 1 | Female | 1 | 0,929 | 0,026 | 0,858 | 0,966 |
| 1 | Male | 1+ | 0,605 | 0,185 | 0,252 | 0,875 |
| 2 | Female | 1 | 0,889 | 0,024 | 0,832 | 0,928 |
| 2 | Male | 1+ | 0,267 | 0,046 | 0,186 | 0,367 |
| 2 | Female | 1 | 0,979 | 0,017 | 0,902 | 0,996 |
| 2 | Male | 1+ | 0,289 | 0,132 | 0,103 | 0,589 |
|  |  |  |  |  |  |  |

**Table S6.** Top 10 best supported models for the effect of ‘Year’ and other covariates on log-transformed daily energy expenditure (DEE, kJ/d) of males pelagic cormorants rearing chicks. The residuals of all top 2ΔAICc models were checked for homoscedasticity and normality. All models but one verified both conditions (i.e. heteroscedasticity was detected in the residuals from model 6, Breusch-Pagan test: BP_5_ = 12.887, *P* = 0.024).

(Int) = Intercept ; B.age = Brood age ; B.size = Brood size ; Tsm = Time since marking ; df = Degree of freedom ; logLik =Log Likelihood, AICc = Akaike Information Criterion corrected for small sample size.

| **Index** | **(Int)** | **Year** | **B.age** | **B.size** | **Tsm** | **B. age×B. size** | **B.age ×Tsm** | **B.age ×Year** | **B.size× Tsm** | **B.size**  **×Year** | **Tsm×Year** | **df** | **logLik** | **AICc** | **ΔAICc** | **weight** |
| --- | --- | --- | --- | --- | --- | --- | --- | --- | --- | --- | --- | --- | --- | --- | --- | --- |
| 1 | 7,268 | + |  |  | -0,012 |  |  |  |  |  |  | 6 | 30,18 | -46,72 | 0,00 | 0,19 |
| 2 | 7,438 | + | -0,013 | -0,046 | -0,045 | 0,005 | 0,002 |  |  |  |  | 10 | 35,36 | -46,03 | 0,69 | 0,14 |
| 3 | 7,181 | + |  | 0,029 | -0,012 |  |  |  |  |  |  | 7 | 31,13 | -46,01 | 0,71 | 0,13 |
| 4 | 7,225 | + |  |  |  |  |  |  |  |  |  | 5 | 28,51 | -45,87 | 0,85 | 0,13 |
| 5 | 7,139 | + |  | 0,029 |  |  |  |  |  |  |  | 6 | 29,37 | -45,09 | 1,63 | 0,08 |
| 6 | 7,231 | + | 0,003 |  | -0,012 |  |  |  |  |  |  | 7 | 30,60 | -44,97 | 1,75 | 0,08 |
| 7 | 7,299 | + | -0,007 | -0,034 | -0,011 | 0,004 |  |  |  |  |  | 9 | 33,23 | -44,71 | 2,01 | 0,07 |
| 8 | 7,184 | + | 0,003 |  |  |  |  |  |  |  |  | 6 | 29,05 | -44,45 | 2,27 | 0,06 |
| 9 | 7,138 | + | 0,003 | 0,030 | -0,012 |  |  |  |  |  |  | 8 | 31,62 | -44,31 | 2,41 | 0,06 |
| 10 | 7,309 | + | -0,001 |  | -0,036 |  | 0,001 |  |  |  |  | 8 | 31,61 | -44,29 | 2,43 | 0,06 |
|  |  |  |  |  |  |  |  |  |  |  |  |  |  |  |  |  |

**Table S7.** Top 10 best supported models for the effect of ‘Year’ and other covariates on the daily proportion of time spent flying (%) by males pelagic cormorants rearing chicks. The residuals of all top 2ΔAICc models were checked for homoscedasticity and normality. All models verified both conditions.

(Int) = Intercept ; B.age = Brood age ; B.size = Brood size ; Tsm = Time since marking ; df = Degree of freedom ; logLik =Log Likelihood, AICc = Akaike Information Criterion corrected for small sample size.

| **Index** | **(Int)** | **Year** | **B.age** | **B.size** | **Tsm** | **B. age**  **×B. size** | **B.age**  **×Tsm** | **B.age**  **×Year** | **B.size**  **×Tsm** | **B.size**  **×Year** | **Tsm**  **×Year** | **df** | **logLik** | **AICc** | **ΔAICc** | **weight** |
| --- | --- | --- | --- | --- | --- | --- | --- | --- | --- | --- | --- | --- | --- | --- | --- | --- |
| 1 | 3.928 | + |  |  |  |  |  |  |  |  |  | 5 | -83.33 | 177.81 | 0.00 | 0.32 |
| 2 | 4.094 | + |  |  | -0.046 |  |  |  |  |  |  | 6 | -82.82 | 179.29 | 1.48 | 0.15 |
| 3 | 3.690 | + | 0.018 |  |  |  |  |  |  |  |  | 6 | -82.95 | 179.54 | 1.73 | 0.14 |
| 4 | 3.724 | + |  | 0.068 |  |  |  |  |  |  |  | 6 | -83.23 | 180.10 | 2.29 | 0.10 |
| 5 | 4.520 | + | -0.011 |  | -0.246 |  | 0.010 |  |  |  |  | 8 | -81.06 | 181.05 | 3.24 | 0.06 |
| 6 | 3.866 | + | 0.017 |  | -0.043 |  |  |  |  |  |  | 7 | -82.50 | 181.24 | 3.44 | 0.06 |
| 7 | 3.885 | + |  | 0.070 | -0.046 |  |  |  |  |  |  | 7 | -82.71 | 181.67 | 3.86 | 0.05 |
| 8 | 2.955 |  |  | 0.261 |  |  |  |  |  |  |  | 3 | -87.65 | 181.75 | 3.94 | 0.04 |
| 9 | 3.461 | + | 0.019 | 0.074 |  |  |  |  |  |  |  | 7 | -82.83 | 181.89 | 4.09 | 0.04 |
| 10 | 3.163 |  |  | 0.264 | -0.062 |  |  |  |  |  |  | 4 | -86.85 | 182.45 | 4.64 | 0.03 |
|  |  |  |  |  |  |  |  |  |  |  |  |  |  |  |  |  |

**Table S8.** Top 10 best supported models for the effect of ‘Year’ and other covariates on the daily proportion of time spent diving (%) by males pelagic cormorants rearing chicks. The residuals of all top 2ΔAICc models were checked for homoscedasticity and normality. All models but one verified both conditions (i.e. heteroscedasticity was detected in the residuals from model 3, Breusch-Pagan test: BP_3_= 10.022 , *P* = 0.018).

(Int) = Intercept ; B.age = Brood age ; B.size = Brood size ; Tsm = Time since marking ; df = Degree of freedom ; logLik =Log Likelihood, AICc = Akaike Information Criterion corrected for small sample size.

| **Index** | **(Int)** | **Year** | **B.age** | **B.size** | **Tsm** | **B. age**  **×B. size** | **B.age**  **×Tsm** | **B.age**  **×Year** | **B.size**  **×Tsm** | **B.size**  **×Year** | **Tsm**  **×Year** | **df** | **logLik** | **AICc** | **ΔAICc** | **weight** |
| --- | --- | --- | --- | --- | --- | --- | --- | --- | --- | --- | --- | --- | --- | --- | --- | --- |
| 1 | 1.428 | + |  | -0.088 |  |  |  |  |  | + |  | 9 | -24.40 | 70.55 | 0.00 | 0.25 |
| 2 | 1.589 | + | -0.035 | -0.208 |  | 0.018 |  |  |  |  |  | 8 | -26.14 | 71.22 | 0.67 | 0.18 |
| 3 | 1.164 | + |  |  |  |  |  |  |  |  |  | 5 | -30.58 | 72.31 | 1.76 | 0.10 |
| 4 | 1.660 | + | -0.035 | -0.204 | -0.020 | 0.018 |  |  |  |  |  | 9 | -25.44 | 72.63 | 2.07 | 0.09 |
| 5 | 1.464 | + |  | -0.082 | -0.015 |  |  |  |  | + |  | 10 | -24.01 | 72.71 | 2.16 | 0.09 |
| 6 | 1.969 | + | -0.051 | -0.231 | -0.096 | 0.020 | 0.004 |  |  |  |  | 10 | -24.07 | 72.83 | 2.27 | 0.08 |
| 7 | 1.245 | + |  |  | -0.022 |  |  |  |  |  |  | 6 | -29.83 | 73.32 | 2.76 | 0.06 |
| 8 | 1.451 | + | -0.001 | -0.091 |  |  |  |  |  | + |  | 10 | -24.39 | 73.46 | 2.91 | 0.06 |
| 9 | 0.972 | + |  | 0.064 |  |  |  |  |  |  |  | 6 | -30.03 | 73.70 | 3.15 | 0.05 |
| 10 | 1.098 | + | 0.005 |  |  |  |  |  |  |  |  | 6 | -30.40 | 74.45 | 3.89 | 0.04 |
|  |  |  |  |  |  |  |  |  |  |  |  |  |  |  |  |  |

**Table S9.** Top 10 best supported models for the effect of ‘Year’ and other covariates on the daily proportion of time spent resting on top of the water (in %) by males pelagic cormorants rearing chicks. The dependent variable (% of time spent resting on top of the water) was square transformed to ensure that all models within 2ΔAICc of the best ranked model verified both homoscedasticity and normality.

(Int) = Intercept ; B.age = Brood age ; B.size = Brood size ; Tsm = Time since marking ; df = Degree of freedom ; logLik =Log Likelihood, AICc = Akaike Information Criterion corrected for small sample size.

| **Index** | **(Int)** | **Year** | **B.age** | **B.size** | **Tsm** | **B. age**  **×B. size** | **B.age**  **×Tsm** | **B.age**  **×Year** | **B.size**  **×Tsm** | **B.size**  **×Year** | **Tsm**  **×Year** | **df** | **logLik** | **AICc** | **ΔAICc** | **weight** |
| --- | --- | --- | --- | --- | --- | --- | --- | --- | --- | --- | --- | --- | --- | --- | --- | --- |
| 1 | -29.336 | + | 7.873 |  |  |  |  |  |  |  |  | 6 | -370.81 | 755.27 | 0.00 | 0.41 |
| 2 | 63.364 | + | 3.758 |  | -29.228 |  | 1.453 |  |  |  |  | 8 | -369.15 | 757.23 | 1.97 | 0.15 |
| 3 | -36.388 | + | 7.938 |  | 1.720 |  |  |  |  |  |  | 7 | -370.78 | 757.79 | 2.52 | 0.12 |
| 4 | -32.406 | + | 7.880 | 0.995 |  |  |  |  |  |  |  | 7 | -370.81 | 757.86 | 2.59 | 0.11 |
| 5 | 72.412 | + |  |  |  |  |  |  |  |  |  | 5 | -374.04 | 759.23 | 3.96 | 0.06 |
| 6 | 52.264 | + | 3.751 | 3.861 | -29.475 |  | 1.464 |  |  |  |  | 9 | -369.13 | 760.01 | 4.75 | 0.04 |
| 7 | -76.224 | + | 10.449 | 18.300 |  | -1.159 |  |  |  |  |  | 8 | -370.70 | 760.34 | 5.07 | 0.03 |
| 8 | -39.253 | + | 7.944 | 0.933 | 1.717 |  |  |  |  |  |  | 8 | -370.77 | 760.49 | 5.22 | 0.03 |
| 9 | 58.937 | + | 1.043 |  |  |  |  | + |  |  |  | 9 | -369.50 | 760.74 | 5.48 | 0.03 |
| 10 | 156.980 | + | 2.184 | -36.777 | -59.253 |  | 1.896 |  | 10.943 |  |  | 10 | -368.12 | 760.93 | 5.66 | 0.02 |
|  |  |  |  |  |  |  |  |  |  |  |  |  |  |  |  |  |

**Table S10.** Top 10 best supported models for the effect of ‘Year’ and other covariates on the daily proportion of time spent resting on land (either at the colony or on others roosting sites, in %) by males pelagic cormorants rearing chicks. The residuals of all top 2ΔAICc models were checked for homoscedasticity and normality. All models verified both conditions.

(Int) = Intercept ; B.age = Brood age ; B.size = Brood size ; Tsm = Time since marking ; df = Degree of freedom ; logLik =Log Likelihood, AICc = Akaike Information Criterion corrected for small sample size.

| **Index** | **(Int)** | **Year** | **B.age** | **B.size** | **Tsm** | **B. age**  **×B. size** | **B.age**  **×Tsm** | **B.age**  **×Year** | **B.size**  **×Tsm** | **B.size**  **×Year** | **Tsm**  **×Year** | **df** | **logLik** | **AICc** | **ΔAICc** | **weight** |
| --- | --- | --- | --- | --- | --- | --- | --- | --- | --- | --- | --- | --- | --- | --- | --- | --- |
| 1 | 88.793 | + | -0.332 |  |  |  |  |  |  |  |  | 6 | -194.44 | 402.52 | 0.00 | 0.35 |
| 2 | 91.110 | + | -0.337 | -0.751 |  |  |  |  |  |  |  | 7 | -194.17 | 404.59 | 2.06 | 0.13 |
| 3 | 87.874 | + | -0.324 |  | 0.224 |  |  |  |  |  |  | 7 | -194.18 | 404.60 | 2.08 | 0.12 |
| 4 | 84.500 | + |  |  |  |  |  |  |  |  |  | 5 | -196.98 | 405.12 | 2.60 | 0.10 |
| 5 | 84.285 | + | -0.173 |  | 1.337 |  | -0.052 |  |  |  |  | 8 | -193.26 | 405.47 | 2.94 | 0.08 |
| 6 | 85.653 | + | -0.017 | 1.404 |  | -0.144 |  |  |  |  |  | 8 | -193.43 | 405.79 | 3.27 | 0.07 |
| 7 | 90.206 | + | -0.329 | -0.760 | 0.227 |  |  |  |  |  |  | 8 | -193.91 | 406.76 | 4.23 | 0.04 |
| 8 | 83.483 | + |  |  | 0.281 |  |  |  |  |  |  | 6 | -196.61 | 406.87 | 4.34 | 0.04 |
| 9 | 80.461 | + | -0.077 | 1.585 | 3.191 |  | -0.081 |  | -0.661 |  |  | 10 | -191.28 | 407.24 | 4.72 | 0.03 |
| 10 | 86.416 | + |  | -0.639 |  |  |  |  |  |  |  | 6 | -196.81 | 407.26 | 4.74 | 0.03 |
|  |  |  |  |  |  |  |  |  |  |  |  |  |  |  |  |  |

1. **Figures**

**
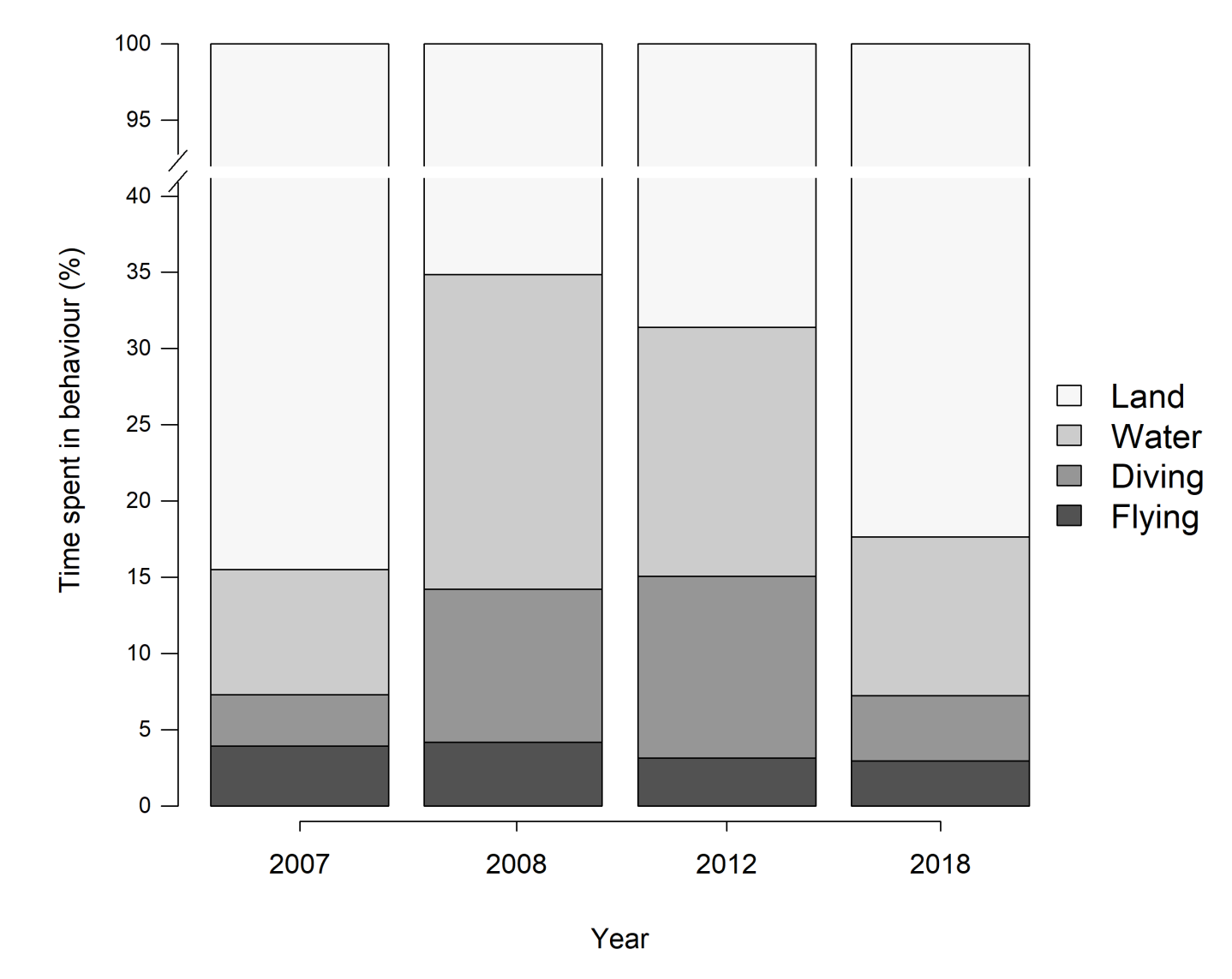
**

**Figure S1:** Comparison of time spent in each of the 4 main behaviours displayed by chick rearing pelagic cormorants in 2007, 2008, 2012 and 2018 (flying, diving, resting on water, and resting on land, either at the colony or on other roosting sites). Pairwise comparisons among years showed that Time spent diving was significantly longer in 2008 and 2012 (9.71 ± 1.28 % and 16.35 ± 3.01 %, respectively) compared to 2007 and 2018 (3.29 ± 0.44 % and 3.62 ± 0.45 %, respectively; *P*<0.05, all others *P*>0.05), matching the pattern observed for DEE. Time spent flying did not match this pattern and was significantly longer only in 2008 (4.20 ± 0.29 %) compared to 2012 and 2018 (3.13 ± 0.23 % and 2.94 ± 0.33 %, respectively; *P*<0.05, all others *P*>0.05). Pelagic cormorants spent less time resting on land in 2008 (63.6 ± 2.06 %) compared to 2007 and 2018 (81.6 ± 2.32 % and 80.1 ± 2.30 %, respectively), and in 2012 (71.8 ± 2.06 %) compared to 2007 (*P*<0.05, all others *P*>0.05). Finally, they spent more time resting on the water in 2008 (21.8 ± 1.42 %) than in other years (2007: 10.4 ± 1.59 %; 2012: 13.9 ± 1.41 %; 2018: 12.3 ± 1.58 %; *P*<0.05, all others *P*>0.05). For each behaviour, the model used for inter-year comparisons was the one with the lowest AICc (Tables S7 to S10).

**
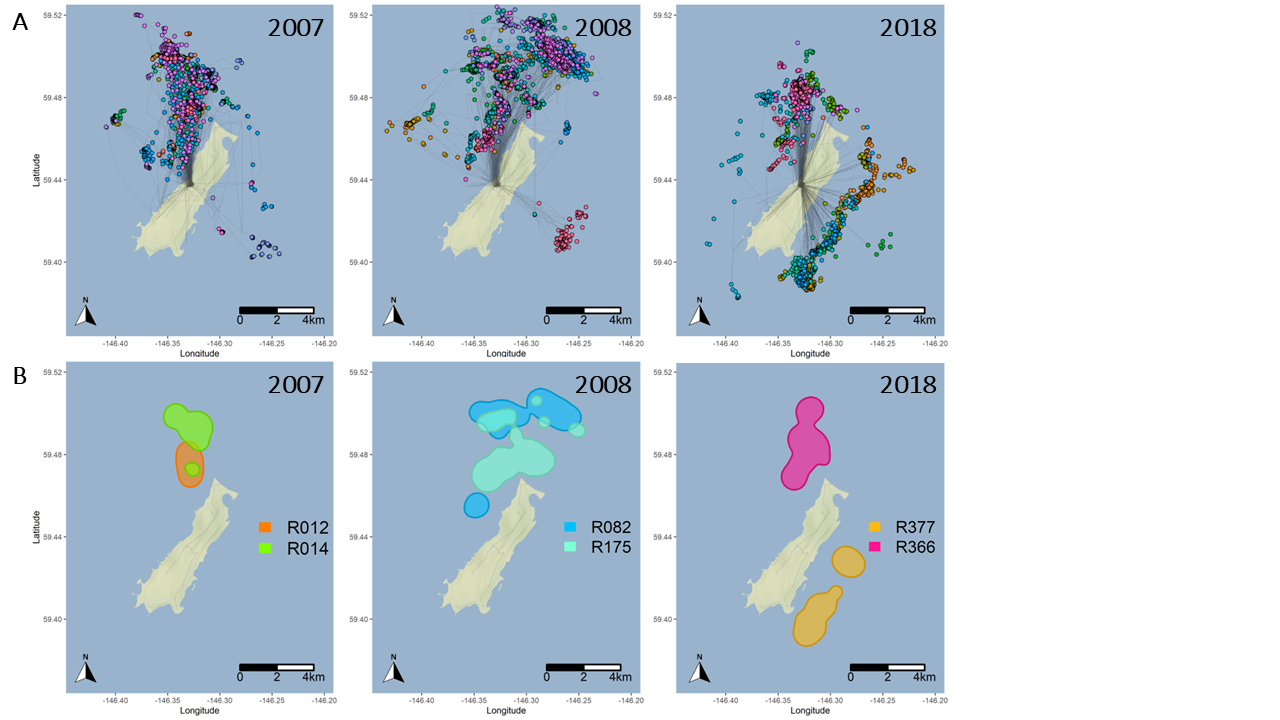
**

**Figure S2:** Inter-annual variation in foraging locations of chick-rearing pelagic cormorants at Middleton Island, AK. **A)** Foraging locations (dots, one color per individual per year) and trips (thin black lines) are displayed for all individuals for which matching GPS and TDR data was available, regardless of duration. The number of tracks varies among years, as different numbers of birds were marked in each year (_n2007_ = 22, _n2008_ = 21, _n2018_ = 18). **B)** Foraging areas (95% utilization distributions) of two randomly picked individuals in each year. The population scale diversification of foraging areas in 2018 compared to 2007-2008 could have indicated a shift in resources distribution and predictability, especially if linked to a decrease in individual foraging site fidelity (IFSF) at the individual level. This hypothesis was tested by calculating an index of foraging site fidelity per individual per year, and then comparing the values among years (2007, 2008, 2018). For this analysis, GPS and dive data were initially processed as in the DEE analysis. Single foraging trips for each individual were then isolated using R package “track2KBA” (Beal et al. 2021). As the focus was on foraging locations, the GPS (sampling interval: 60 to 330 seconds) and dive (sampling interval: 1 or 3 seconds depending on the year) datasets were then homogenized by assigning the closest in time GPS coordinates available from the GPS record to each dive datapoint. All records were further homogenized to keep one datapoint every 3 seconds. Finally, only datapoints indicating a dive deeper than 1m were kept, and trips for which there were less than 15 data points were excluded from the analysis. Only birds with at least 2 complete trips were kept for the remainder of the analysis. Once this dataset of foraging locations had been obtained, we used kernel density analysis to calculate 95% utilization distribution for each complete foraging trip, using R package “adehabitatHR” (Calenge 2006). The smoothing parameter (h) was chosen by averaging the h values obtained with the *ad hoc* method for bivariate normal kernels (href, as implemented in R package “adehabitatHR”) for all trips and all individuals. The resulting value (h=149) was consistent with values for another marine cormorant species (Morgan *et al.* 2019), and we used this fixed value of h for the analysis, over 0.1 km2 grid cells.

We then measured the spatial similarity of the areas used in different trips for each individual by using Bhattacharyya's affinity (Fieberg & Kochanny 2005). This index of similarity can range from 0 to 1 and high values indicate high spatial similarity. Values of similarity were obtained for each pair of trips within an individual, and we averaged those values so to obtain a single value per individual per year. This value was considered to reflect the level of foraging site fidelity of each individual within a given season (hereafter termed IFSF). We found no evidence for changes in IFSF among years (binomial GLM, χ²_2_ = 0.607 , *P =* 0.738), therefore providing little support for the hypothesis that food predictability was lower at the end of the study. Nonetheless the diversification pattern observed at the population level could still be explained by changes in prey distribution, for example if individuals switched their preferred foraging areas, while conserving a high degree of fidelity to these new locations.

**II-Box**

**Box 1: Extracting activity rates from GPS-Pressure data**

Activity rates were extracted from GPS-Pressure data using R version 3.6.2. In order to limit misinterpretation of behaviour, we cleansed the dataset by discarding successive data points in-between which the time interval lasted more than 330 seconds, or less than 5 seconds. We also discarded successive data points that resulted in the individual’s speed exceeding a threshold of 50 meters per second (m/s). As a result of this cleansing, the time over which behaviour was effectively recorded was not necessarily continuous. We thus defined an indicator of measurement quality (percentage of the last 24 - or 48, or 72 - real hours that was correctly sampled by the device) and set the threshold for inclusion at 60%. With this threshold, there was no significant effect of measurement quality on DEE or on the time spent in each of the four predefined behaviours (flying, diving, resting on land, resting on the water, linear regression models, all *P >* 0.05) and we thus concluded that tracking devices performance did not hamper our ability to record time activity budgets reliably.

An individual was considered flying when its speed exceeded the threshold of 6.5 m/s during a given time interval. When speed was below 6.5 m/s, an individual was either considered to be “resting on land” (when positioned inside a polygon encompassing the whole Middleton island) or to be “in the water” (when located outside the polygon, i.e. in open water). The pressure data was used to partition the time spent “in the water” between time spent “diving” and time spent “resting on the water”. Individuals were considered diving when pressure exceeded 1.1 bar (corresponding to the pressure under 1 meter of sea water). The overall time spent by an individual in a given behaviour (i.e. either “flying”, “diving”, “resting on land”, or “resting on the water”) was calculated as the sum of all the time intervals an individual spent in this behaviour.

To ensure that all records contained comparable proportions of nighttime and daytime, we restricted the analysis to 24 hours multiples (24, 48 or 72 hours). For birds with 24 to 47 hours of data, we used only the last 24h (n=36). For birds with 48-71 hours of data we used only the last 48 hours (n=25), and for bird with 72-95 hours of data we used only the last 72 hours (n=10). For example, in the case of a bird with 28 hours of data, we would have discarded the first 4 hours and used the last 24 hours in the analysis.

**III-References**

Beal, M., Oppel, S., Handley, J., Pearmain, E. J., Morera‐Pujol, V., Carneiro, A. P., ... & Dias, M. P. (2021). track2KBA: An R package for identifying important sites for biodiversity from tracking data. Methods in Ecology and Evolution, 12(12), 2372-2378.

Calenge, C. (2006). The package “adehabitat” for the R software: a tool for the analysis of space and habitat use by animals. Ecological modelling, 197(3-4), 516-519.

Fieberg, J., & Kochanny, C. O. (2005). Quantifying home‐range overlap: The importance of the utilization distribution. *The Journal of Wildlife Management*, *69*(4), 1346-1359.

Morgan, E. A., Hassall, C., Redfern, C. P., Bevan, R. M., & Hamer, K. C. (2019). Individuality of foraging behaviour in a short-ranging benthic marine predator: incidence and implications. Marine Ecology Progress Series, 609, 209-219.
