# Supplementary figures and images for: Survival costs of reproduction are independent of energy costs in a seabird, the pelagic cormorant
Source: Ecol Evol. 2024 Jul 23;14(7):e11414. doi: 10.1002/ece3.11414 (PMC11264352; doi:10.1002/ece3.11414)

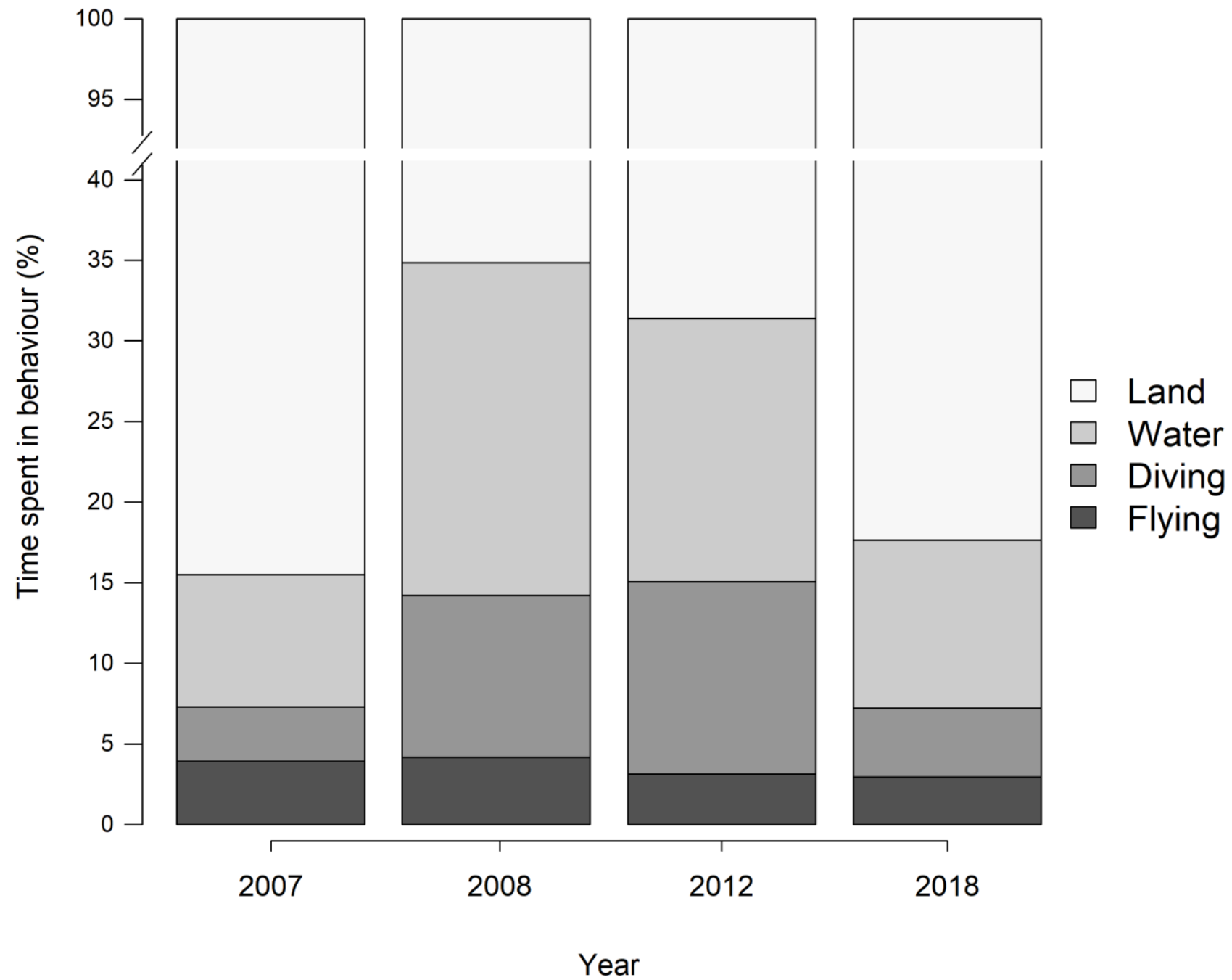

Supplement: Supplementary file 1 — Appendix S1. [file ECE3-14-e11414-s001.zip › FigureS1.pdf]

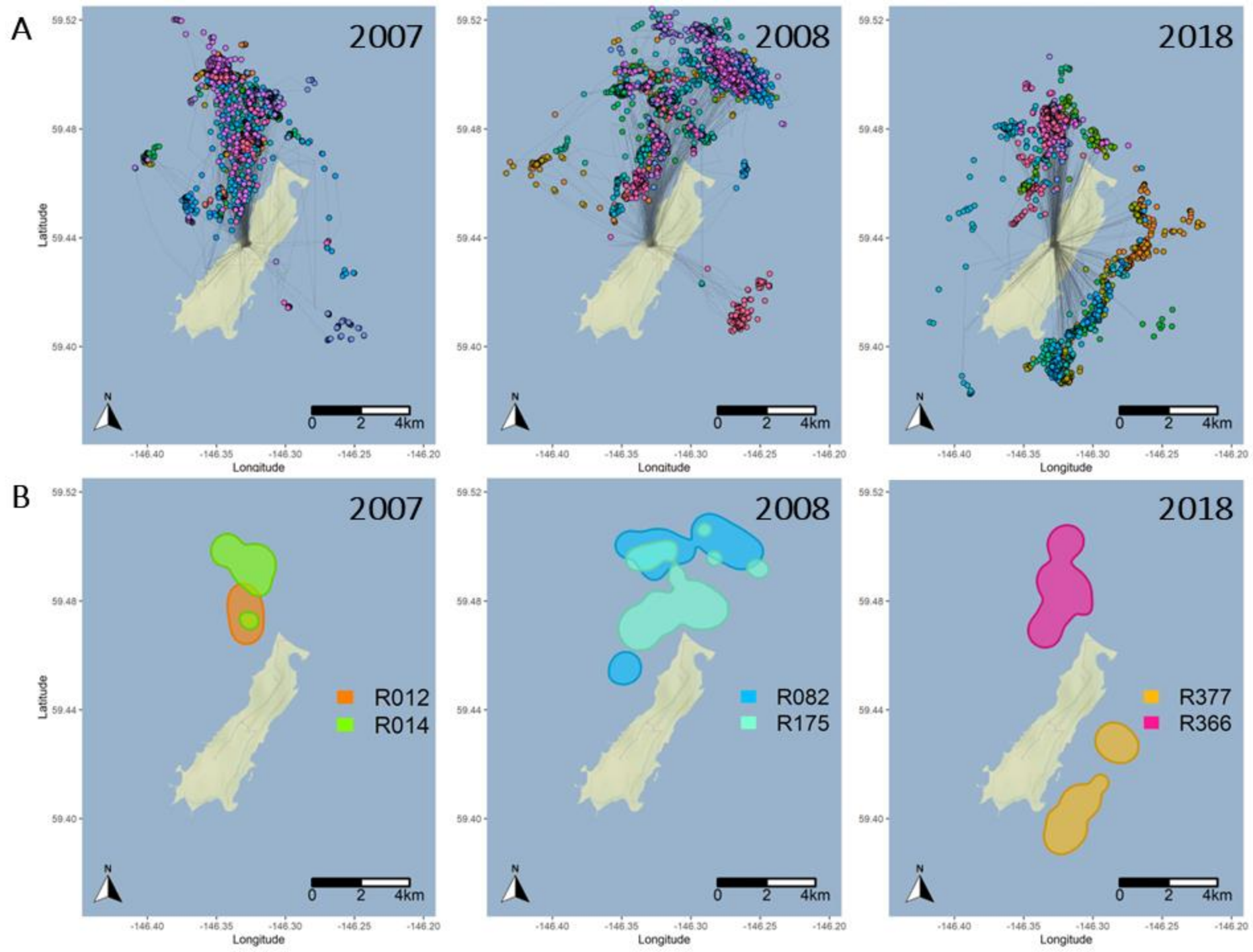

Supplement: Supplementary file 1 — Appendix S1. [file ECE3-14-e11414-s001.zip › FigureS2.pdf]
